# Exploring the mechanism of action of the combination of cinnamon and motherwort in the treatment of benign prostatic hyperplasia: A network pharmacology study

**DOI:** 10.1097/MD.0000000000037902

**Published:** 2024-04-26

**Authors:** Jiutian Yang, Dongyue Ma, Ziwei Zhao, Jun Guo, Kai Ren, Fu Wang, Jun Guo

**Affiliations:** a Graduate School of Beijing University of Chinese Medicine, Chaoyang District, Beijing, China; b Department of Andrology, Xiyuan Hospital of China Academy of Chinese Medical Sciences, Haidian District, Beijing, China; c Graduate School of China Academy of Chinese Medical Sciences, Dongzhimen, Dongcheng District, Beijing, China.

**Keywords:** benign prostatic hyperplasia, Network pharmacology, Traditional Chinese Medicine

## Abstract

Cinnamon and motherwort are traditional Chinese medicines and are often combined to treat benign prostatic hyperplasia; however, the specific therapeutic mechanisms involved remain unclear. Therefore, in this study, we applied a network pharmacology approach to investigate the potential mechanisms of action of the drug pair cinnamon and motherwort (PCM) for the treatment of benign prostatic hyperplasia. Relevant targets for the use of PCM to treat benign prostatic hyperplasia were obtained through databases. Protein–protein interactions were then identified by the STRING database and core targets were screened. Enrichment analysis was conducted through the Metascape platform. Finally, molecular docking experiments were carried out to evaluate the affinity between the target proteins and ligands of PCM. We identified 22 active ingredients in PCM, 315 corresponding targets and 130 effective targets of PCM for the treatment of benign prostatic hyperplasia. These targets were related to the PI3K-Akt, MAPK, FoxO, TNF, and IL-17 signaling pathways. Network pharmacology was used to identify the effective components and action targets of PCM. We also identified potential mechanisms of action for PCM in the treatment of benign prostatic hyperplasia. Our results provide a foundation for expanding the clinical application of PCM and provide new ideas and directions for further research on the mechanisms of action of PCM and its components for the treatment of benign prostatic hyperplasia.

## 1. Introduction

Benign prostatic hyperplasia (BPH) is a nonmalignant growth or enlargement of prostate tissue that is a common cause of lower urinary tract symptoms in men. The clinical symptoms of BPH include frequent urination, urgency, painful urination, and difficulty urinating. With the aggravation of global aging, the incidence rate of BPH is increasing year by year.^[1]^ The incidence of this disease has increased over recent years and studies have shown that the prevalence of BPH is >50% in men aged 60 years and can exceed 80% by 80 years.^[2]^ BPH is a progressive disease, which may lead to urinary retention, recurrent hematuria, recurrent urinary tract infection, bladder stones, renal insufficiency and other complications, which not only significantly reduce the quality of life of patients, but also cause harm to the physical and mental health of patients, and lead to a series of serious social impact and economic burden. The pathogenesis of this disease remains unclear, although mainstream medicine believes that it may be associated with aging and the status of testicular function.^[3]^ At present, the main pharmacological treatment of BPH in the clinic involves α1-blockers, 5α-reductase inhibitors and phosphodiesterase 5 inhibitors. These drugs can significantly improve lower urinary tract symptoms in patients with BPH, but they also have certain safety issues. Alpha 1-blockers have side effects such as dizziness, orthostatic hypotension, and retrograde ejaculation; 5α-reductase inhibitors have side effects such as decreased libido and erectile dysfunction; phosphodiesterase 5 inhibitors have side effects such as flushing, gastroesophageal reflux, headache, dyspepsia, back pain, and nasal congestion.

Traditional Chinese Medicine (TCM) believes that BPH is closely related to the kidneys and bladder. As age increases, the human body is prone to kidney Qi deficiency, which can lead to unfavorable gasification of the kidneys and bladder. In addition, the local dampness and heat accumulation in the prostate can cause symptoms such as frequent urination, urgency, painful urination, and yellowing of urine during the acute phase. The increase in prostate volume is also related to blood stasis. Prostate hyperplasia caused by Qi stagnation and blood stasis needs to promote blood circulation and remove blood stasis. TCM tends to treat based on syndrome differentiation, using medication more accurately according to each individual’s physical characteristics. TCM has outstanding advantages in shortening the course of treatment and reducing the economic burden on patients. Usually, TCM does not use a single herb to treat a disease; rather, TCM is applied as a synergistic multi-drug combination. Cinnamon and motherwort are often used as a drug combination to treat BPH. Cinnamon is the dried bark of *Cinnamomum cassia* Presl (Camphoraceae) while motherwort is the fresh or dried above-ground part of *Leonurus japonicus* Houttuyn (Labiatae). The combination of these 2 drugs can treat BPH caused by deficiency of kidney Qi, unfavorable Qi-transformation in the bladder and blood stasis. However, the mechanism of action of this drug combination in the treatment of BPH remains unclear; furthermore, the overall therapeutic effect of this drug combination has rarely been reported.

**Figure 1.**
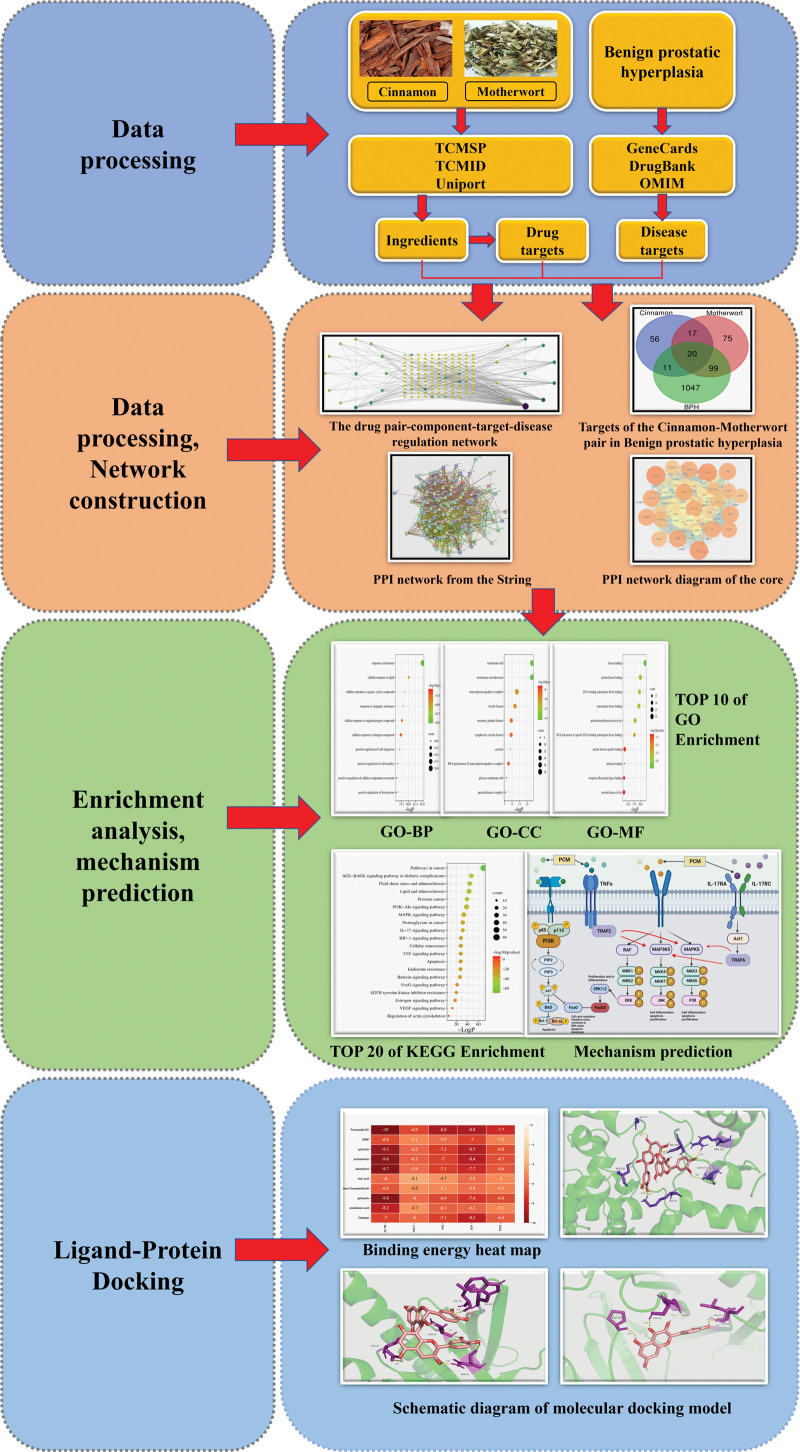
Flow diagram showing the technical strategy used in this study. The dark blue part is the screening method and process of drugs and disease-related targets; the orange part is the drawing of target-related network diagrams and drug-disease target-related Wayne diagram; the light green part is the prediction diagram of GO, KEGG and signal pathway related to benign prostatic hyperplasia; the light blue part is the flow chart of hydrogen bond binding energy and molecular docking. KEGG = Kyoto Encyclopedia of Genes and Genomes.

Network pharmacology is an emerging discipline that is increasingly being applied to investigate the pharmacological mechanisms of action of herbal medicines.^[4–6]^ This method integrates existing pharmacological methods, high-throughput bioinformatics and high-end data software and can provide a comprehensive analysis of the mechanisms of action of specific herbal medicines.^[7,8]^ In this study, we investigated the molecular mechanisms of pair cinnamon and motherwort (PCM) for the treatment of BPH by applying a network pharmacology approach. The flowchart of the technical strategy in this study is shown in Figure 1.

## 2. Materials and methods

### 2.1. Screening of PCM active ingredients and potential targets

We used the Traditional Chinese Medicine Systematic Pharmacology Database and Analysis Platform (TCMSP, https://old.tcmsp-e.com/tcmsp.php)^[9]^ and used the keywords “cinnamon” and “motherwort” to find their chemical composition. Next, we identified the corresponding Molecule IDs (Mol IDs). The pharmacokinetic absorption, distribution, metabolism, and excretion parameters (ADME) were then identified for the active ingredients of cinnamon and motherwort by applying an oral availability ≥ 30% and drug-like properties ≥ 0.18 for motherwort and ≥ 0.10 for cinnamon; then, we obtained the corresponding Molecule IDs (Mol IDs). The active ingredients of cinnamon and motherwort were further supplemented by the comprehensive database of Chinese Medicine (TCMID – Database Commons [cncb.ac.cn])^[10]^ and by screening the related literature. Finally, we supplemented the compounds contained in PubMed and CNKI according to the relevant literature reports.^[11]^ After screening, the validated human protein and gene information was downloaded from the Uniport database (https://www.uniprot.org)^[12]^ and combined with information from the DrugBank database (https://www.drugbank.ca)^[13]^ to standardize the collected protein target information into uniform gene names.

### 2.2. BPH-related target screening

Using “benign prostatic hyperplasia” and “benign prostatic hypertrophy” as keywords, we used the GeneCards database (https://www.genecards.org)^[14]^ with a median value greater than the relevance score as a screening criterion to identify target genes with high relevance to BPH and searched the OMIM database (http://www.omim.org)^[15]^ and DrugBank database (https://www.drugbank.ca) for further BPH-related targets. BPH-related targets were obtained by merging disease database targets and removing duplicate entries. After screening, the Uniprot database (https://www.uniprot.org) was used to standardize the names of the BPH-related targets.

### 2.3. Construction of an “herbal-component-target-disease” network

Common targets were obtained by taking the intersection of the action targets of PCM active ingredients with the BPH targets using the Draw Venn Diagram online tool (http://bioinformatics.psb.ugent.be/webtools/Venn/) to draw a Venn diagram. Cytoscape 3.7.1 software^[16]^ was then used to construct a “herbal-component-target-disease” network; different colors and shapes were used to mark and visualize the contents and interrelationships of each component. The greater the degree of connectivity, the more biological functions are involved and the higher the importance; this allowed us to identify the important components of PCM that play a role in the treatment of BPH.

### 2.4. Construction of a protein-protein interaction (PPI) network and the screening of core targets

The active ingredients of PCM and the common intersection targets for BPH were submitted to the String 11.5 platform (https://string-db.org)^[17]^ to construct a PPI network with the biological species set to “*Homo sapiens*” and the remaining parameters as default settings. The output data were imported into CytoScape 3.7.1 software for network analysis and network mapping, and the main action targets were further screened according to the magnitude of the degree value.

### 2.5. Biological enrichment analysis

Data relating to the main targets of PCM for BPH were imported into the Metascape platform (http://metascape.org/gp/index.html)^[18]^ for Gene Ontology (GO) functional analysis and Kyoto Encyclopedia of Genes and Genomes (KEGG) of Genes and Genomes (KEGG) pathway enrichment analysis. GO analysis included biological process (BP), CC (cellular component) and MF (molecular function). Results were saved and visualized using the Micro-biotics online tool (http://www.bioinformatics.com.cn).

### 2.6. Molecular docking validation

The top 5 targets in the drug-disease target PPI network were downloaded from the RCSB PDB database (https://rcsb.org),^[19]^ exported to PDB format and dehydrated. Then, we removed the unwanted ligands using Pymol software and exported data in PDBQT format after hydrogenation using Autodock software. Next, we downloaded the structural files of the active ingredients with the top 10 degree values in the “herbal-component-target-disease” network for the treatment of BPH from the TCM Systematic Pharmacology Database and Analysis Platform (TCMSP, https://tcmspw.com/tcmsp.php). Structure files (in Mol2 format) of the top 10 active ingredients in the “degree-value” network were exported in PDBQT format files after defining torsion using Autodock software. Then, we opened the PDBQT files of receptor proteins and ligand small molecules in Autodock software, generated docking boxes and adjusted the parameters; we set exhaustiveness to 10, docked the 5 core targets to the 10 active ingredients by Autodock vina and analyzed the docking results obtained. Binding strength and activity were evaluated based on the binding energy and the number of hydrogen bonds generated.

### 2.7. Ethical statement

This study does not include studies of human subjects, human data, or human tissues or animals. All data is obtained from the database, so ethical certification is not required.

## 3. Results

### 3.1. Active ingredients and targets of the cinnamon-motherwort drug pair

Following absorption, distribution, metabolism, and excretion screening, literature supplementation and the deletion of chemical components relating to nonhuman targets, a total of 12 active ingredients of cinnamon and 10 active ingredients of motherwort were obtained, totaling 22 active ingredients. A total of 159 targets of cinnamon and 334 targets of motherwort were further obtained and 315 targets were obtained after combining and deleting duplicate and invalid items (Tables 1 and 2).

**Table 1:** Active ingredients and corresponding targets of cinnamon.

MOLID	Active Ingredients	Target
MOL000131	EIC	PTGS1, PTGS2, RXRA, NCOA2, SLC6A2, IGHG1, GABRA2, GABRA1, TRPV1, CHRM1, CHRM2, GABRA6
MOL000208	()-Aromadendrene	CHRM3, CHRM2, CHRM1, ADH1C
MOL000266	beta-Cubebene	CHRM1, CHRM2, GABRA1, PTGS2, CHRNA2, SLC6A2
MOL002697	junipene	CHRM3, CHRM2, GABRA1, GABRA2, ADRA1B, CHRNA2
MOL003522	()-Sativene	CHRM3, CHRM1, PTGS2, GABRA2, GABRA3, CHRM2, ADRA1B CHRNA2, GABRA1, CHRNA7, NCOA2
MOL003538	()-Ledene	NCOA2, CHRM3, CHRM1
MOL002003	(-)-Caryophyllene oxide	CHRM3, CHRM1, PTGS2, ACHE, CHRM2, ADRA1B, GABRA1 DPP4, GABRA6
MOL000057	DIBP	CHRM3, CHRM1, SLC6A2, SLC6A3, ADRB2, SLC6A4, GABRA1, CHRM2, HTR2A, PGR, NR3C2, NR3C1, NCOA2, RXRA
MOL000612	(-)-alpha-cedrene	CHRM3, PTGS2, GABRA2, RXRA, GABRA1, NCOA2, CHRM1, ADH1B, ADH1C
MOL000675	oleic acid	PTGS1, NCOA2, PTGS2, ADH1B, ADH1C, ADH1A, PRSS3, RXRA, PLAU, SOD1, CAT, TEP1, EDN1, ERBB2, PPARG, LPL, GAP43, SERPINE1, BDNF, HMGCR, MPO, PPARA, PPARD, CRP, PON1, INS, PLG, FABP1, RBP2, ENPEP, UCP2, SOAT1, CCK, CITED1, NTRK2, PDX1, SLC2A2, PAM, UCP3, CETP, PYY, DNPEP
MOL000004	Procyanidin B1	ACTB, AHR, ATP5A1, ATP5B, ATP5C1, CBR1, CDK6, CEBPB, CSNK2A1, CSNK2B, CYP1B1, DHFRL1, DNMT1, EIF3F, ESR1, ESR2, HCK, HIBCH, HSP90A, HSPA2, JAK1, NQO2, NR1I2, PIK3CG, PIM1, RUVBL2, SF3B3, SHBG, STK17B, UBA1, UGT3A1
MOL000991	cinnamaldehyde	ADH1B, RELA, IRF3, PTGS2, NFKBIA, TLR4, NFE2L2, C5AR, TRPV1, TRPV4, TXNRD1, IFNB1

**Table 2:** Active ingredients and corresponding targets of motherwort.

MOLID	Active Ingredients	Target
MOL001418	galeopsin	CHRM1, ADRB1, PTGS2, ADRA2A, RXRA, ACHE, SLC6A2, CHRM2, ADRA2B, SLC6A3, ADRB2, SLC6A4, MAOB
MOL001420	ZINC04073977	PGR, PTGS2, GABRA1, ADH1C
MOL001421	preleoheterin	CHRM3, CHRM1, PTGS2, PDE3A, SLC6A2, ADRA1B, ADRB2, SLC6A4, F10, HSP90A, CALM1
MOL001422	iso-preleoheterin	PTGS1, CHRM1, PTGS2, RXRA, SLC6A2, GABRA1, GRIA2
MOL000098	quercetin	PTGS1, AR, PPARG, PTGS2, HSP90A, PIK3CG, NCOA2, DPP4, AKR1B1, PRSS1, TOP2, KCNH2, SCN5A, F10, ADRB2, MMP3, F7, NOS3, RXRA, ACHE, GABRA1, MAOB, RELA, EGFR, AKT1, VEGFA, CCND1, BCL2, BCL2L1, FOS, CDKN1A, EIF6, BAX, CASP9, PLAU, MMP2, MMP9, MAPK1, IL10, EGF, RB1, TNF, JUN, IL6, AHSA1, CASP3, TP53, ELK1, NFKBIA, POR, ODC1, XDH, CASP8, TOP1, RAF1, SOD1, PRKCA, MMP1, HIF1A, STAT1, RUNX1T1, HSPA5, ERBB2, PPARG, ACACA, HMOX1, CYP3A4, CYP1A2, CAV1, MYC, F3, GJA1, CYP1A1, ICAM1, IL1B, CCL2, SELE, VCAM1, PTGER3, CXCL8, PRKCB, BIRC5, DUOX2, NOS3, HSPB1, TGFB1, SULT1E1, MGAM, IL2, NR1I2, CYP1B1, CCNB1, PLAT, THBD, SERPINE1, COL1A1, IFNG, ALOX5, PTEN, IL1A, MPO, TOP2A, NCF1, ABCG2, HAS2, GSTP1, NFE2L2, NQO1, PARP1, AHR, PSMD3, SLC2A4, COL3A1, CXCL11, CXCL2, DCAF5, NR1I3, CHEK2, INSR, CLDN4, PPARA, PPARD, HSF1, CRP, CXCL10, CHUK, SPP1, RUNX2, RASSF1, E2F1, E2F2, ACP3, CTSD, IGFBP3, IGF2, CD40LG, IRF1, ERBB3, PON1, DIO1, PCOLCE, NPEPPS, HK2, NKX3-1, RASA1, GSTM1, GSTM2
MOL001439	arachidonic acid	PTGS1, PTGS2, RXRA, TRPV1, RXRG, SLC6A2, RELA, CCND1, MAPK1, EGF, CASP3, PPARG, G6PD, TNFRSF1A, PRKCB, PECAM1, NOS3, ALOX5, PLA2G4A, PTEN, SELP, PTGES, GLB1, ALDH2, ABCA1, ALDH3A1, UCP2, C1R, CETP, ABCG1, ABCC4, KCNK10, TNFRSF1B, PTGES2, KCNK2, COL1A2
MOL000354	isorhamnetin	NOS2, PTGS1, ESR1, AR, PTGS2, ESR2, MAPK14, GSK3B, SP90A, PIK3CG, PRSS1, PIM1, CCNA2, NCOA2, CALM1PYGM, PPARD, CHEK1, AKR1B1, NCOA1, F7, NOS3, ACHE, GABRA1, MAOB, GRIA2, RELA, XDH, NCF1, OLR1
MOL000422	kaempferol	NOS2, PTGS1, AR, PTGS2, HSP90A, PIK3CG, NCOA2, DPP4, PRSS1, PGR, CHRM1, NOS3, GABRA2, ACHE, SLC6A2, CHRM2, ADRA1B, GABRA1, TOP2, F7, CALM1, RELA, IKBKB, AKT1, BCL2, BAX, TNF, JUN, AHSA1, CASP3, MAPK8, XDH, MMP1, STAT1, PPARG, HMOX1, CYP3A4, CYP1A2, CYP1A1, ICAM1, SELE, VCAM1, NR1I2, CYP1B1, ALOX5, HAS2, GSTP1, AHR, PSMD3, SLC2A4, NR1I3, INSR, DIO1, PPP3CA, GSTM1, GSTM2, AKR1C3, SLPI
MOL005573	Genkwanin	GABRA1, GABRA2, GABRA3, GABRA4, GABRA5, GABRA6, GABRG1, GABRG2, GABRG3, MTTP, SHBG, SOAT1 , SOAT2
MOL002563	galangin	NOS2, PTGS1, AR, PPARG, PTGS2, PDE3A, DPP4, HSP90A, PIK3CG, CHEK1, BCL2, CYP1A1, CCND3, GSTP1, AHR

### 3.2. BPH-related targets

The highest relevance score was 113.12 and the lowest value was 0.24 (due to the excessive number of targets); 974 targets were retained after filtering by the median relevance score according to experience. In total, 209 BPH-related targets were obtained from the OMIM database and 40 BPH-related targets were obtained from the Drugbank database. The BPH-related targets obtained from each database were combined and duplicates were removed; 1177 BPH-related targets remained (Table S1, Supplemental Digital Content, http://links.lww.com/MD/M242).

### 3.3. Construction of a “herbal-component-target-disease” network for cinnamon and motherwort for BPH treatment

**Figure 2.**
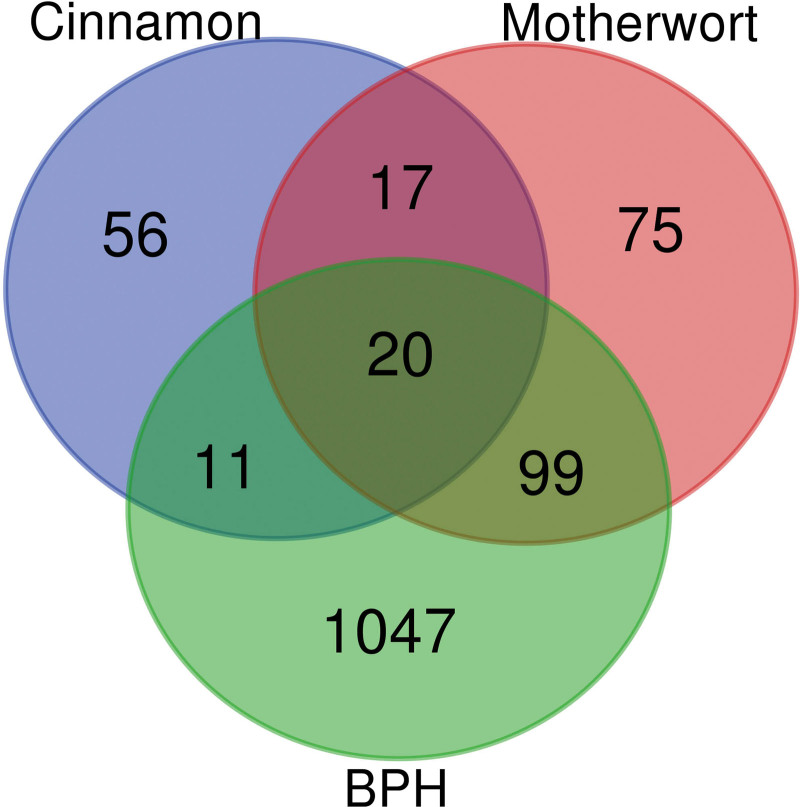
Targets for PCM in the treatment of benign prostatic hyperplasia. The number in the purple circle is the number of unique targets for cinnamon; the number in the pink circle is the number of unique targets for motherwort, and the number in the green circle is the number of unique targets for benign prostatic hyperplasia. The number in the middle is the number of common targets for both. PCM = pair cinnamon and motherwort.

The obtained PCM action targets were intersected with BPH disease targets using the Draw Venn Diagram online tool (http://bioinformatics.psb.ugent.be/webtools/Venn/) to obtain a total of 130 potential targets for PCM treatment of BPH (Fig. 2).

**Figure 3.**
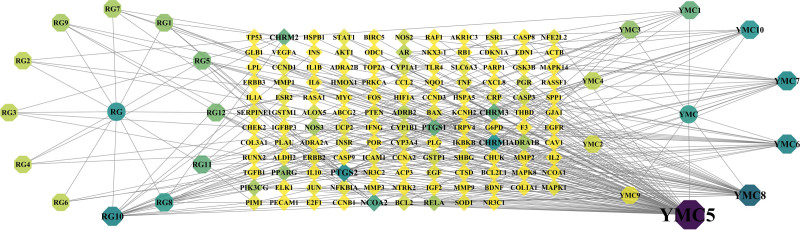
Image showing the drug pair-component-target-disease regulation network. RG and YMC represent the traditional Chinese medicines cinnamon and motherwort, respectively. RG1-12 represents cinnamon-related compounds and YMC1-10 represents motherwort-related compounds. The middle rhomboid node is the target protein; the darker the color, the larger the node and the higher the corresponding degree value.

Cytoscape 3.7.1 software was used to construct and analyze the “herbal-component-target” network (Fig. 3); a total of 154 nodes and 278 edges were identified. The circle represents the Chinese herbal medicines, the square octagon represents the main active ingredients and the diamond represents the common targets; the degree value is indicated by the color shade, node size and transparency. The top 10 active ingredients in terms of degree value are shown in Table 3. The higher the degree value of the active ingredient, the more the ingredient interacts with the target in BPH. This data shows that the main active ingredients of PCM for the treatment of BPH are quercetin, kaempferol, oleic acid, arachidonic acid, isorhamnetin, galangin, diisobutyl phthalate, proanthocyanidin B1, fenugreek diterpene, and cinnamaldehyde.

**Table 3:** Main active ingredients of cinnamon and motherwort.

MOLID	Active ingredients	Degree	Betweenness centrality	Closeness centrality	Neighborhood connectivity
MOL000098	Quercetin	96	0.71441104	0.59765625	1.9893617
MOL000422	Kaempferol	34	0.141649	0.41351351	4.08823529
MOL000675	Oleic acid	17	0.09786169	0.37684729	3.94117647
MOL001439	Arachidonic acid	17	0.06897366	0.36428571	4
MOL000354	Isorhamnetin	16	0.08728467	0.37135922	4.5625
MOL002563	Galangin	11	0.02010427	0.35747664	5.54545455
MOL000057	DIBP	10	0.03945366	0.35253456	5.8
MOL000004	Procyanidin B1	8	0.03224339	0.34459459	3.625
MOL001418	Galeopsin	8	0.0368413	0.35915493	6.75
MOL000991	Trans-cinnamaldehyde	7	0.03159747	0.35915493	5.57142857

### 3.4. The “herbal-disease” target PPI network

**Figure 4.**
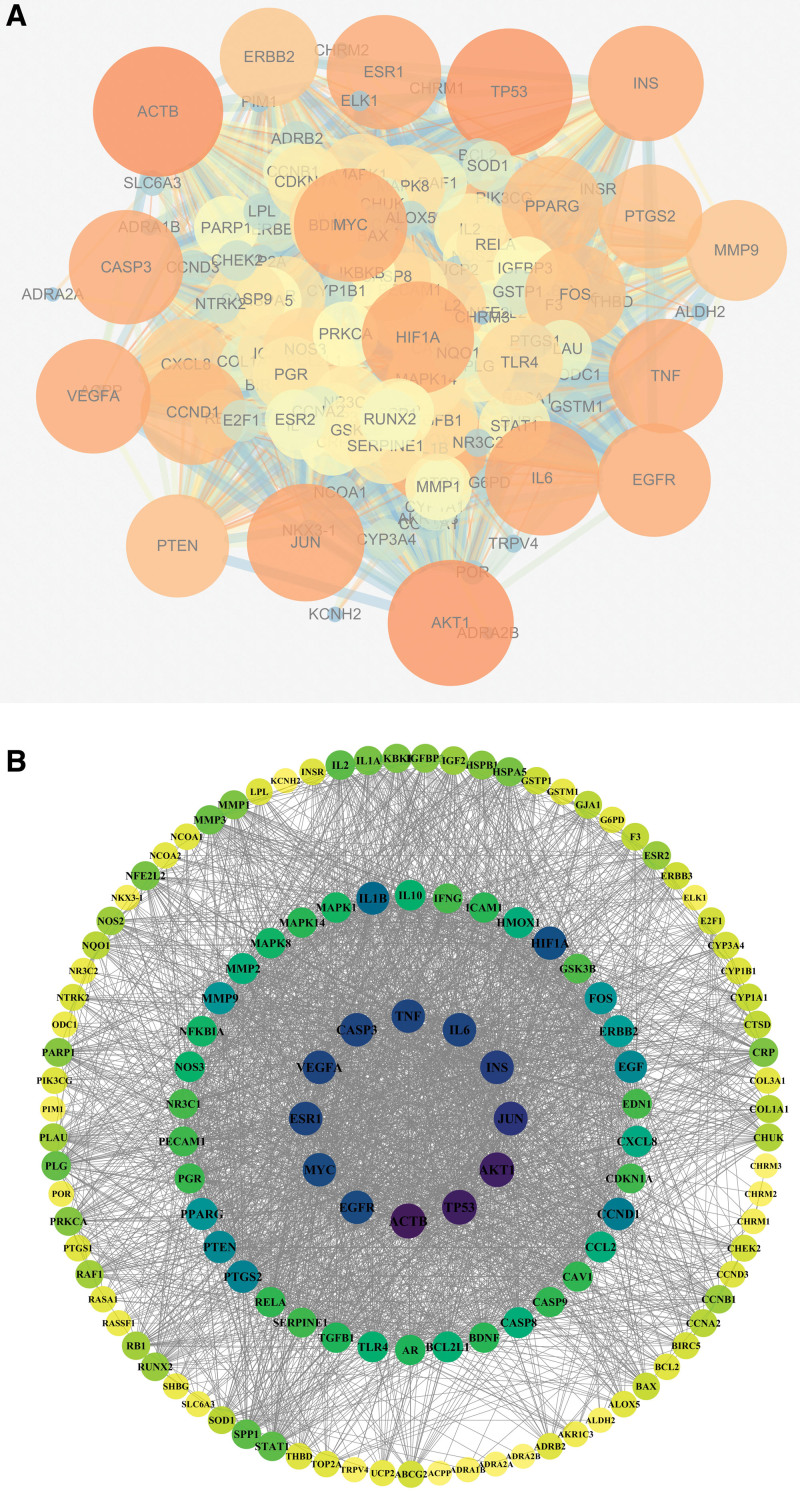
The “herbal-disease” target network diagram: (A) the “herbal-disease” target network diagram generated by Cytoscape software, (B) the “herbal-disease” target PPI network generated by Cytoscape software.

The common targets were submitted to the String 11.5 platform and a PPI network was derived (Fig. 4A). Cytoscape 3.7.1 software was then used to construct the PPI network for the common targets of PCM for BPH treatment. The topology of the PPI network was derived by using the “Analyze Network” function in Cytoscape 3.7.1 software to derive the degree, closeness centrality, neighborhood connectivity and betweenness centrality. The network was sorted by degree value and plotted in Figure 4B. The size of degree value is shown by color shade, node size and transparency. A higher degree value indicates a stronger interaction between the target and other targets and the top 20 targets with degree and other important association values are shown in Table 4: beta actin (ACTB), tumor protein P53 (TP53), serine/threonine protein kinase 1 (AKT1), Jun proto-oncogene (AP-1 transcription factor subunit, JUN), insulin (INS), Caspase 3 (CASP3), vascular endothelial growth factor A (VEGFA), estrogen receptor 1 (ESR1), interleukin-6 (IL-6), tumor necrosis factor (TNF), MYC proto-oncogene (MYC), epidermal growth factor receptor (EGFR), hypoxia-inducible factor 1α subunit (HIF1A), interleukin-1β (IL-1B), cell cycle protein D1 (CCND1), prostaglandin-endoperoxide synthase 2 (PTGS2), and recombinant human epidermal growth factor (EGF), protein tyrosine phosphatase gene (PTEN), peroxisome proliferator-activated receptor gamma (PPARG) and Fos proto-oncogene (AP-1 transcription factor subunit, FOS).

**Table 4:** Characteristic parameters of the network nodes for the main targets of PCM for the treatment of BPH.

Target	Degree	Betweenness centrality	Closeness centrality	Neighborhood connectivity
ACTB	105	0.047097	0.847682	48.98095238
TP53	102	0.032581	0.831169	49.98039216
AKT1	101	0.032731	0.820513	50.00990099
JUN	93	0.032163	0.785276	52.60215054
INS	91	0.021023	0.775758	53.0989011
CASP3	90	0.01567	0.771084	54.2
VEGFA	90	0.015737	0.771084	54.21111111
ESR1	90	0.027195	0.766467	50.66666667
IL6	90	0.03346	0.771084	53.2
TNF	90	0.01926	0.771084	53.44444444
MYC	89	0.021356	0.761905	52.31460674
EGFR	89	0.021427	0.766467	52.86516854
HIF1A	88	0.01649	0.761905	54.54545455
IL1B	84	0.012495	0.744186	55.25
CCND1	81	0.013966	0.723164	54.32098765
PTGS2	80	0.012018	0.727273	56.9625
EGF	79	0.009352	0.723164	57.30379747
PTEN	79	0.01426	0.719101	54.59493671
PPARG	77	0.014801	0.715084	55.50649351
FOS	77	0.017244	0.711111	56.53246753

### 3.5. GO and KEGG enrichment analysis

**Figure 5.**
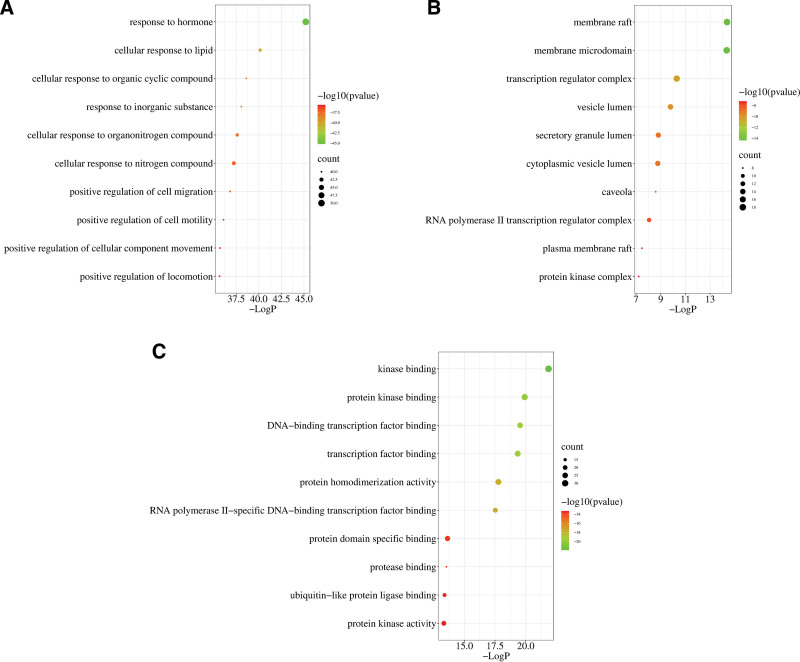
Top 10 terms from GO enrichment: (A) the top 10 terms for GO enrichment (BP), (B) the top 10 terms for GO enrichment (CC), and (C) the top 10 terms for GO enrichment (MF). The *y*-axis represents the top 10 BP/CC/MF terms and the x-axis represents the enrichment factors. GO = Gene Ontology.

**Figure 6.**
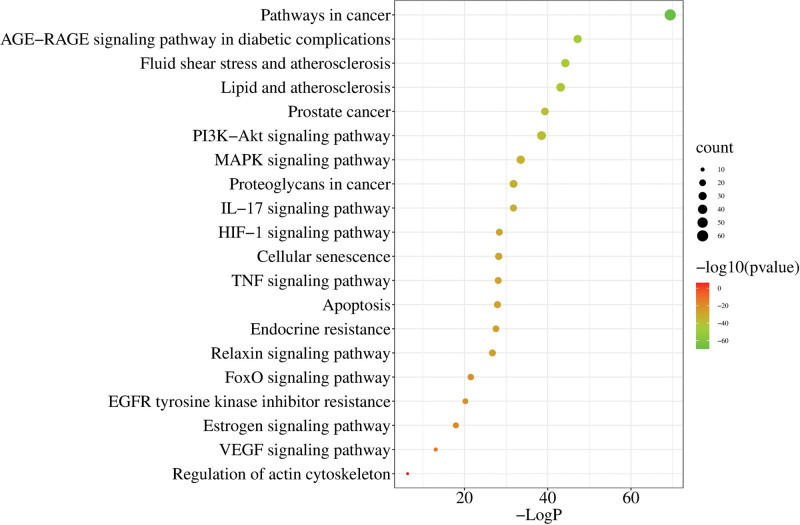
Top 20 KEGG enrichment terms. The y-axis the top 20 KEGG terms and the *x*-axis represents the enrichment factors. KEGG = Kyoto Encyclopedia of Genes and Genomes.

The targets of PCM for BPH were imported into the Metascape platform for GO and KEGG enrichment analysis and the species of the database and gene set were limited to “*Homo sapiens*”; threshold was set at *P* < .01. The metabolic pathways of apparently unrelated diseases were excluded from the KEGG enrichment results. The data were exported and visualized using the Micro-biology online tool. The main BPs involved in PCM included response to hormones, inorganic substances, lipids, organic nitrogen compounds, organic cyclic compounds, positive regulation of cell migration, cell motility, and CC movement. The main CCs of PCM action included membrane rafts, membrane microregions, transcriptional regulatory complexes, vesicle lumen, secretory granule lumen, cytoplasmic vesicle lumen, niche, RNA polymerase II transcriptional regulatory complexes, plasma membrane rafts, and protein kinase complexes. The main molecular functions of PCM action were kinase binding, protein kinase binding, DNA binding and transcription factor binding, transcription factor binding, protein homodimerization activity, RNA polymerase II-specific DNA binding and transcription factor binding, protein structural domain-specific binding, protease binding, ubiquitin-like protein ligase binding and protein kinase activity (Fig. 5 and Table S2, Supplemental Digital Content, http://links.lww.com/MD/M243). The main metabolic pathways that PCM acts on for the treatment of BPH include cancer-related pathways, inflammation and infection-related pathways, prostate cancer, proteoglycans in cancer, cellular senescence, apoptosis, endocrine resistance, relaxin-related signaling pathways, the PI3K-Akt signaling pathway, the MAPK signaling pathway, the IL-17 signaling pathway, the chemo-oncogenic-receptor activation pathway, the HIF-1 signaling pathway and the TNF signaling pathway. The top 20 most relevant signaling pathways were selected and included in the network diagram analysis (Fig. 6 and Table S3, Supplemental Digital Content, http://links.lww.com/MD/M244). The *P*-value data related to GO and KEGG are shown in Table S4, Supplemental Digital Content, http://links.lww.com/MD/M245.

### 3.6. Molecular docking results

**Figure 7.**
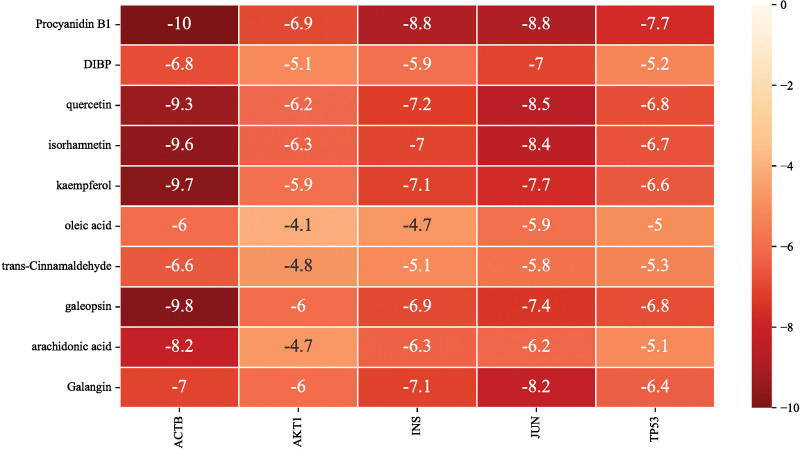
Thermogram showing molecular docking binding energies. Values represent the minimum binding energy value; the lower the binding energy, the darker the color. The *y*-axis represents the top 10 chemical compounds and the *x*-axis represents the top 5 targets.

**Figure 8.**
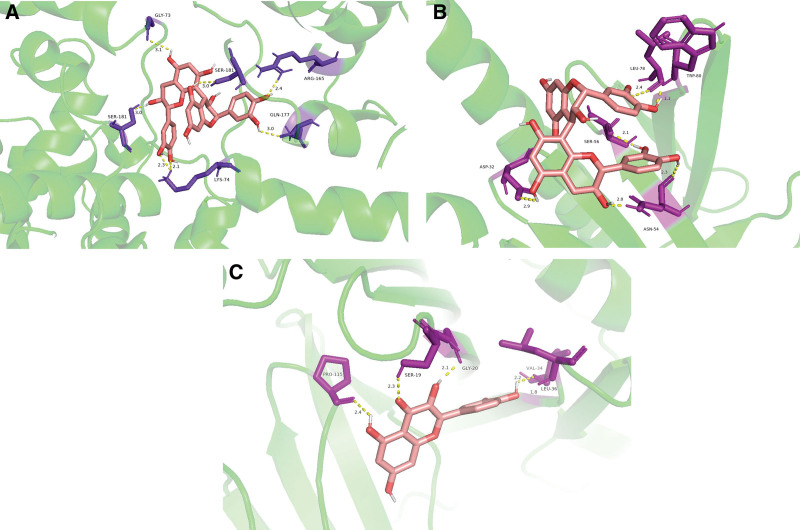
Schematic diagram of the molecular docking model. (A) Schematic diagram of docking between ACTB and Procyanidin B1. (B) Schematic diagram of docking between AKT1 and Procyanidin B1. (C) Schematic diagram of docking between INS and quercetin.

The top 5 targets (in terms of degree-value for the PPI network) were molecularly docked to the active components of PCM; the receptors were ACTB (PDB ID: 6ict), TP53 (PDB ID: 2k8f), AKT1 (PDB ID: 1unq), JUN (PDB ID: 1s9k) and INS (PDB ID: 1jk8). The binding strength and activity were evaluated based on the binding energy and the number of hydrogen bonds generated. The lower the binding energy and the higher the number of hydrogen bonds, the more stable the binding conformation and the higher the binding activity of the receptor protein and the small molecule ligands (Fig. 7). The values marked in the graph refer to the minimum binding energy. Selected binding conformations of interactions with ≥ 5 hydrogen bonds were visualized using Pymol (Fig. 8).

## 4. Discussion

With the continuous aging of the population, BPH has become an important public health issue, dragging down the economy and individual quality of life.^[20,21]^ Although there are available treatment methods, their effectiveness and potential side effects are limited, which makes the task of researchers to develop more effective solutions to meet urgent needs.^[22]^ According to the theory of Qi and blood in TCM, Qi deficiency should be tonified and blood stasis should be invigorated. Cinnamon and motherwort, as a combination of commonly used drugs in TCM clinics, are often used to treat BPH with kidney Qi deficiency and blood stasis blockage.

**Figure 9.**
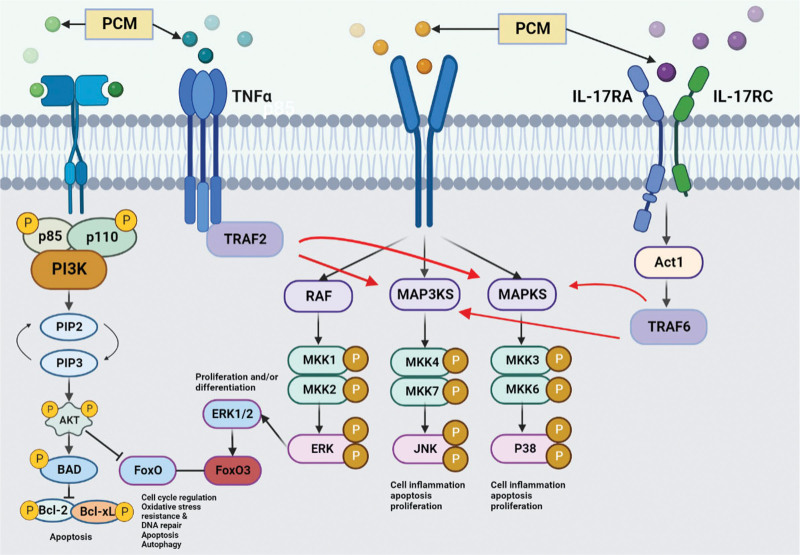
Schematic diagram of the signaling pathways associated with benign prostatic hyperplasia.

In this study, we used network pharmacology to investigate the potential therapeutic mechanism of PCM in BPH. Through database screening, 22 main active ingredients and 315 target genes were identified in PCM. After intersecting with BPH related target genes, 130 potential drug targets for treating BPH were obtained, including ACTB, TP53, AKT1, JUN, INS, EGFR, EGF, VEGFA, IL-6, CASP3, ESR1, etc. Subsequently, enrichment analysis was performed on 130 target genes, and the results showed that their BPs include cell proliferation and apoptosis, inflammation, response to growth factors, oxidative stress, etc The positive regulation of cell migration and movement mainly involves the PI3K-Akt signaling pathway, MAPK signaling pathway, FoxO signaling pathway, TNF signaling pathway, IL-17 signaling pathway, and other pathways, as shown in Figure 9.

Research has shown that the imbalance between proliferation and apoptosis of prostate epithelial and stromal cells, excessive cell proliferation, and inhibition of apoptosis are important reasons for the occurrence and development of BPH.^[23–25]^ Therefore, restoring the balance between proliferation and apoptosis is crucial for the treatment of BPH. Phosphatidylinositol-3 kinases (PI3Ks) are a family of lipoprotein kinases that regulate various intracellular metabolic activities. PI3K produces the second messenger phosphatidylinositol-di/trisphosphate (PIP3).^[26]^ Protein kinase B (Akt) is a serine/threonine protein kinase that leads to an increase in cell growth and survival by phosphorylating key molecules in downstream pathways.^[27]^ PIP3 further activates Akt to regulate the function of multiple substrates, thus leading to the phosphorylation of the pro-apoptotic protein BAD; this prevented the binding of BAD to the antiapoptotic molecules Bclxl and Bcl2, thereby inhibiting apoptosis.^[28]^ Another study found that overexpression of the PI3K/AKT pathway leads to a reduction in apoptosis in BPH, thereby leading to an increase in prostate volume.^[29]^ MAPK is an evolutionarily conserved family of proline-directed serine/threonine kinases that consists of a three-component kinase cascade: the extracellular signal-regulated kinase (ERK 1/2) cascade, the stress-activated protein kinase/c-Jun N-terminal kinase (SAPK1/JNK) cascade, and the p38-MAPK cascade. JNK and p38 exert similar functions and are associated with cell inflammation, apoptosis and growth. ERK is mainly associated with cell growth and differentiation. Previous studies found that hyperplasia of the prostate epithelium and stroma in BPH patients was associated with all 3 of these MAPK cascades.^[30–32]^ As a transcription factor downstream of AKT, the Fox O signaling pathway similarly promotes apoptosis while inhibiting the cell cycle progression and autophagy.^[33]^ A previous study found that downregulation of the FoxO signaling pathway may be related to the pathogenesis of BPH.^[34]^

Inflammation is believed to be closely related to the onset of BPH.^[35–37]^ The TNF signaling pathway is considered a key inflammatory pathway that promotes the occurrence of BPH. TNF is a collective name for 2 classes of related cytokines: TNFα and TNFβ; these exert a range of biological functions such as antiviral effects, the promotion of cell proliferation and differentiation, and plays an important regulatory role in inflammation, immune response and various pathophysiological processes. A study found that in BPH, immunoreaction to TNFα decreased as compared with that of normal prostates, while immunoreactions to both TNFα receptors increased.^[38]^ Another previous study found that TNF antagonists significantly reduced epithelial hyperplasia, NF-κB activation, and macrophage-mediated inflammation in prostate tissues and reduced the incidence of BPH.^[39]^ The IL-17 family consists of 6 structurally related cytokines: IL-17A, IL-17B, IL-17C, IL-17D, IL-17E (IL-25) and IL-17F. IL-17 induces downstream genes *via* the activation of NF-kB and MAPK. Previous researchers found that prostatitis is strongly associated with an increased risk of worsening lower urinary tract symptoms, a risk of urinary retention and the need for surgery.^[40]^ Overexpression of the IL-17 signaling pathway pro-inflammatory factor IL-17 stimulates a multifold increase in IL-6 and IL-8, thus promoting the growth of the BPH endothelial matrix.^[35,41]^ In addition, IL-6, IL-8 and IL-17 may perpetuate chronic immune response in BPH and induce fibromuscular growth by an autocrine or paracrine loop or via induction of COX-2 expression. Immune reaction may be activated via Toll-like receptor signaling and mediated by macrophages and T cells. Conversely, anti-inflammatory factors such as macrophage inhibitory cytokine-1 decreased in symptomatic BPH tissues.^[42]^

In addition to drugs such as α-blockers and PCM, clinical treatment of BPH also includes a number of other phytomedicines and non-pharmacological therapies. Currently, commonly used botanicals include Serenoa repens, Pygeum africanum, Urtica dioica and Cucurbita pepo.^[43]^ Systematic reviews have suggested that both saw palmetto and Pygeum africanum provide modest improvement in urinary symptoms and flow.^[44–46]^ Non-pharmacologic therapies include electroacupuncture, prostatic microwave thermotherapy, water vapor thermal therapy, prostatic urethral lift, prostatic arterial embolization, temporary implantable nitinol device and transurethral resection of the prostate. A study found that electroacupuncture can reduce the International Prostate Symptom Score, maximum urinary flow rate, and post-void residual volume in patients with BPH. It is safe and effective for clinical use in the treatment of BPH.^[47]^ Another study found that symptoms and quality of life for water vapor thermal therapy, prostatic urethral lift and prostatic arterial embolization appeared similar to those for transurethral resection of the prostate, whereas transurethral resection of the prostate was found to have the most clinically significant improvement in flow rate. Prostatic microwave thermotherapy was less efficacious than transurethral resection of the prostate.^[48]^

Network pharmacology methods also have some limitations in TCM research. Firstly, the functionality of database software is weak. The accuracy and comprehensiveness of target information in the database need to be improved, the correlation between drug components and their targets between databases is low, the protein-protein interaction network needs to be further improved, and the stability of the website still needs to be strengthened; Additionally, the accuracy of target prediction is not high. The targets obtained by network pharmacology of traditional Chinese medicine compound formulas are mainly obtained by superimposing the predicted targets of various single Chinese medicine components. However, the accuracy of the targets obtained by network pharmacology methods is relatively low, because in actual experiments and clinical medication processes, different Chinese medicine preparations, different decoction methods and times can affect the efficacy of drugs. Therefore, the components of traditional Chinese medicine supplements used in experiments or clinical practice are more complex than those of single Chinese medicine components. Deep learning algorithms have a strong ability to extract key features from datasets. In future research, artificial intelligence algorithms can be combined with network pharmacology to develop new network algorithms, analyze and reduce the dimensionality of large-scale data obtained from network pharmacology, and obtain key information. In addition, the construction of any theoretical model cannot be separated from experimental verification, and the same applies to the study of network pharmacology of traditional Chinese medicine. Only by combining network-based computational prediction with experimental confirmation, and conducting biological experiments on the prediction results of network pharmacology can the accuracy and reliability of the prediction results be verified.

## 5. Conclusion

In this study, we utilized network pharmacology and molecular docking methods to investigate the pharmacological mechanism of PCM in treating BPH. Our research findings indicate that PCM counteracts BPH by targeting multiple pathways and targets. Specifically, we propose that the PI3K-Akt, MAPK, FoxO, TNF, and IL-17 signaling pathways are the main mechanisms by which PCM treats BPH. Overall, our research provides valuable theoretical basis for further studying the mechanism of PCM treatment for BPH and its potential clinical applications. Meanwhile, this study has some limitations. Firstly, the active ingredients of PCM are predicted through computer calculations, and the pharmacokinetic characteristics in the human body still need to be detected. More importantly, potential therapeutic targets, BPs, and signaling pathways of PCM have been revealed through network pharmacology, but the results lack relevant experimental validation. Follow-up studies should aim to validate these findings in animal experiments and clinical studies to improve the rationality and scientific basis for the clinical application of PCM.

## Acknowledgments

This work was supported by High-level Traditional Chinese Medicine Key Discipline Construction Project of National Administration of Traditional Chinese Medicine (zyyzdxk-2023238); Youth Realism Project of China Association of Chinese Medicine (No.2023-QNQS-02) and Specialized R&D and Transformation of Medical Institution Preparations and New Chinese Medicines with Intellectual Property Rights of Xiyuan Hospital of China Academy of Chinese Medical Sciences (XYZX0301-13).

## Author contributions

**Conceptualization:** Jiutian Yang, Fu Wang, Jun Guo.

**Data curation:** Jiutian Yang, Dongyue Ma.

**Formal analysis:** Jiutian Yang.

**Funding acquisition:** Fu Wang.

**Investigation:** Jiutian Yang.

**Methodology:** Jiutian Yang.

**Project administration:** Jiutian Yang, Dongyue Ma.

**Resources:** Jiutian Yang, Ziwei Zhao, Jun Guo.

**Software:** Jiutian Yang, Ziwei Zhao, Kai Ren.

**Supervision:** Jiutian Yang.

**Validation:** Jiutian Yang, Jun Guo.

**Visualization:** Jiutian Yang, Ziwei Zhao, Kai Ren.

**Writing – original draft:** Jiutian Yang, Fu Wang.

**Writing – review & editing:** Jiutian Yang, Fu Wang, Jun Guo.

## Supplementary Material








