# Supplementary material for: Exploring the mechanism of action of the combination of cinnamon and motherwort in the treatment of benign prostatic hyperplasia: A network pharmacology study
Source: Medicine (Baltimore). 2024 Apr 26;103(17):e37902. doi: 10.1097/MD.0000000000037902 (PMC11049697; doi:10.1097/MD.0000000000037902)
Supplement: Supplementary file 1 [file medi-103-e37902-s001.docx]

| BPH motherwort cinnamon 20 | BPH motherwort 99 | BPH cinnamon 11 | motherwort cinnamon 17 | motherwort75 | cinnamon 56 | BPH 1047 |
| --- | --- | --- | --- | --- | --- | --- |
| CHRM1 | MMP2 | EDN1 | SLC6A2 | PTGER3 | HSPA2 | CD44 |
| PPARG | HSPB1 | BDNF | SLC6A4 | XDH | CSNK2A1 | IGS1 |
| CRP | PTEN | NR3C1 | RXRA | EIF6 | PIK3CG | INSL3 |
| NFE2L2 | NOS2 | NR3C2 | DPP4 | C1R | NQO2 | ERBB4 |
| ADRA1B | CCNB1 | INS | PON1 | DCAF5 | PYY | KCNMA1 |
| PTGS2 | ALOX5 | NTRK2 | MPO | IRF1 | CDK6 | SPGP |
| ADRB2 | GJA1 | TRPV4 | ADH1C | PCOLCE | ESR2 | SPINK1 |
| UCP2 | COL1A1 | ACTB | GABRA2 | PRKCB | UGT3A1 | MEFV |
| SOD1 | BIRC5 | TLR4 | GABRA6 | CLDN4 | IRF3 | ATRX |
| ERBB2 | ALDH2 | PLG | SOAT1 | ABCG1 | PIM1 | DCP1 |
| RELA | SHBG | LPL | ACHE | ACACA | CITED1 | CYP11A1 |
| PGR | BCL2 |  | GABRA1 | PPP3CA | CHRNA2 | HPN |
| CHRM2 | ADRA2A |  | TRPV1 | ABCA1 | CBR1 | MIR31 |
| SERPINE1 | KCNH2 |  | PPARD | CHEK1 | HSP90A | HSD17B1 |
| NCOA2 | CYP1A1 |  | PPARA | PSMD3 | DNMT1 | NBN |
| CHRM3 | E2F1 |  | CETP | F7 | GAP43 | BID |
| NFKBIA | CASP8 |  | GABRA3 | DUOX2 | CCK | CXCR4 |
| PTGS1 | GSTP1 |  |  | PTGES | PRSS3 | CUBN |
| SLC6A3 | CXCL8 |  |  | CXCL11 | CSNK2B | TSPY1 |
| PLAU | PIM1 |  |  | GABRG1 | HIBCH | IGF1 |
|  | THBD |  |  | MTTP | RUVBL2 | SOS1 |
|  | MAPK14 |  |  | SELE | ATP5C1 | MIR146A |
|  | TNF |  |  | AHR | C5AR | TFM |
|  | RAF1 |  |  | ABCC4 | HMGCR | TGFB2 |
|  | EGF |  |  | NR1I3 | NR1I2 | MIR20A |
|  | IL1A |  |  | KCNK10 | PAM | GAS5 |
|  | CHUK |  |  | ADRB1 | DHFRL1 | RAC1 |
|  | SPP1 |  |  | PLA2G4A | ESR1 | LAMA2 |
|  | CCND1 |  |  | SELP | HCK | HPC4 |
|  | ESR1 |  |  | TNFRSF1A | CEBPB | NTRK1 |
|  | CTSD |  |  | OLR1 | TEP1 | KLK6 |
|  | POR |  |  | SLPI | SF3B3 | ANXA1 |
|  | VEGFA |  |  | NCF1 | ATP5B | GDF15 |
|  | TGFB1 |  |  | HAS2 | FABP1 | CYCS |
|  | MYC |  |  | RUNX1T1 | DNPEP | MMP7 |
|  | CCNA2 |  |  | GABRG3 | PDX1 | EP300 |
|  | GSK3B |  |  | PDE3A | RBP2 | DYT10 |
|  | CCL2 |  |  | MGAM | ENPEP | MITF |
|  | MMP1 |  |  | RXRG | ATP5A1 | HPCX1 |
|  | CCND3 |  |  | SULT1E1 | TXNRD1 | SETD2 |
|  | STAT1 |  |  | KCNK2 | IGHG1 | S100P |
|  | IL6 |  |  | TOP2 | AHR | MMP13 |
|  | CASP3 |  |  | MAOB | ADH1A | DHTR |
|  | PARP1 |  |  | CYP1A2 | UBA1 | SMAX1 |
|  | COL3A1 |  |  | AHSA1 | SHBG | SEC23B |
|  | ABCG2 |  |  | NPEPPS | ADH1B | SERPINB5 |
|  | HMOX1 |  |  | PLAT | CAT | ZEB1 |
|  | PECAM1 |  |  | CXCL2 | JAK1 | ERG |
|  | MMP3 |  |  | ALDH3A1 | SLC2A2 | FAT4 |
|  | ELK1 |  |  | HK2 | HTR2A | SOD2 |
|  | GSTM1 |  |  | F10 | UCP3 | MIR15A |
|  | IL10 |  |  | TNFRSF1B | IFNB1 | SYNE2 |
|  | MAPK1 |  |  | AKR1B1 | CYP1B1 | MSH3 |
|  | IGF2 |  |  | SLC2A4 | STK17B | RNF14 |
|  | ADRA2B |  |  | HSF1 | EIF3F | CD4 |
|  | PIK3CG |  |  | GABRA4 | CHRNA7 | BAK1 |
|  | EGFR |  |  | E2F2 |  | UGT2B17 |
|  | IKBKB |  |  | PTGES2 |  | DSP |
|  | NQO1 |  |  | CXCL10 |  | FGFR1 |
|  | NKX3-1 |  |  | VCAM1 |  | CHRM5 |
|  | IL2 |  |  | HSP90A |  | MET |
|  | ERBB3 |  |  | DIO1 |  | MSMP |
|  | IFNG |  |  | GABRG2 |  | PTK2B |
|  | FOS |  |  | GABRA5 |  | BBC3 |
|  | HSPA5 |  |  | NR1I2 |  | SDHB |
|  | G6PD |  |  | PYGM |  | PCAT29 |
|  | TOP2A |  |  | GSTM2 |  | MAX |
|  | MAPK8 |  |  | CALM1 |  | NGF |
|  | ICAM1 |  |  | COL1A2 |  | SLCO1B3 |
|  | CAV1 |  |  | CD40LG |  | COX14 |
|  | BCL2L1 |  |  | SCN5A |  | MIR200B |
|  | NCOA1 |  |  | GRIA2 |  | NANOG |
|  | CHEK2 |  |  | PRSS1 |  | NRP1 |
|  | HIF1A |  |  | SOAT2 |  | CDH13 |
|  | NOS3 |  |  | TOP1 |  | LEP |
|  | RB1 |  |  |  |  | PKD1 |
|  | ACP3 |  |  |  |  | BRV2 |
|  | RUNX2 |  |  |  |  | FASN |
|  | ODC1 |  |  |  |  | OXT |
|  | TP53 |  |  |  |  | SHC1 |
|  | INSR |  |  |  |  | TRPM8 |
|  | CASP9 |  |  |  |  | HPC15 |
|  | CDKN1A |  |  |  |  | PCAT19 |
|  | RASA1 |  |  |  |  | IRX5 |
|  | AKT1 |  |  |  |  | HSD11B2 |
|  | IL1B |  |  |  |  | BRCA1 |
|  | AKR1C3 |  |  |  |  | HYSP1 |
|  | IGFBP3 |  |  |  |  | CYP2D6 |
|  | F3 |  |  |  |  | ACVRL1 |
|  | GLB1 |  |  |  |  | MECP2 |
|  | JUN |  |  |  |  | EFNB1 |
|  | AR |  |  |  |  | MIR30A |
|  | ESR2 |  |  |  |  | CYP2C19 |
|  | BAX |  |  |  |  | SMO |
|  | PRKCA |  |  |  |  | PKD2 |
|  | RASSF1 |  |  |  |  | BMPR1A |
|  | CYP3A4 |  |  |  |  | PRL |
|  | CYP1B1 |  |  |  |  | TIMP3 |
|  | MMP9 |  |  |  |  | BFH |
|  |  |  |  |  |  | CA21H |
|  |  |  |  |  |  | KRT1 |
|  |  |  |  |  |  | LCAM |
|  |  |  |  |  |  | MIR331 |
|  |  |  |  |  |  | ANXA3 |
|  |  |  |  |  |  | TEK |
|  |  |  |  |  |  | SQSTM1 |
|  |  |  |  |  |  | INTS6 |
|  |  |  |  |  |  | UGT2B7 |
|  |  |  |  |  |  | MIR149 |
|  |  |  |  |  |  | MTHFR |
|  |  |  |  |  |  | PTOV1 |
|  |  |  |  |  |  | PFIC2 |
|  |  |  |  |  |  | PTPN11 |
|  |  |  |  |  |  | SMAD3 |
|  |  |  |  |  |  | ATF3 |
|  |  |  |  |  |  | SSTR2 |
|  |  |  |  |  |  | FOXP3 |
|  |  |  |  |  |  | PLK1 |
|  |  |  |  |  |  | GNAS |
|  |  |  |  |  |  | GHR |
|  |  |  |  |  |  | DYSF |
|  |  |  |  |  |  | ITGB4 |
|  |  |  |  |  |  | MIR148A |
|  |  |  |  |  |  | KRT13 |
|  |  |  |  |  |  | PITA3 |
|  |  |  |  |  |  | DICER1 |
|  |  |  |  |  |  | NCOR2 |
|  |  |  |  |  |  | DRD5 |
|  |  |  |  |  |  | H2AC18 |
|  |  |  |  |  |  | MIR151A |
|  |  |  |  |  |  | FOLH1 |
|  |  |  |  |  |  | MSMB |
|  |  |  |  |  |  | DMTN |
|  |  |  |  |  |  | LGALS1 |
|  |  |  |  |  |  | CASP10 |
|  |  |  |  |  |  | ALK |
|  |  |  |  |  |  | EZH2 |
|  |  |  |  |  |  | TRPV6 |
|  |  |  |  |  |  | PIAS3 |
|  |  |  |  |  |  | MSH6 |
|  |  |  |  |  |  | LFS2 |
|  |  |  |  |  |  | MVCD3 |
|  |  |  |  |  |  | FAS |
|  |  |  |  |  |  | MIR127 |
|  |  |  |  |  |  | ORM1 |
|  |  |  |  |  |  | ARNT |
|  |  |  |  |  |  | HBEGF |
|  |  |  |  |  |  | BFNIS |
|  |  |  |  |  |  | MIR99A |
|  |  |  |  |  |  | CDK1 |
|  |  |  |  |  |  | CDKN1B |
|  |  |  |  |  |  | DEL17q24 |
|  |  |  |  |  |  | PTK2 |
|  |  |  |  |  |  | LOC106099062 |
|  |  |  |  |  |  | RNS4 |
|  |  |  |  |  |  | CYP21A2 |
|  |  |  |  |  |  | STEAP3 |
|  |  |  |  |  |  | MIR223 |
|  |  |  |  |  |  | CSF1 |
|  |  |  |  |  |  | TUBB |
|  |  |  |  |  |  | REST |
|  |  |  |  |  |  | TNFSF10 |
|  |  |  |  |  |  | BFIS4 |
|  |  |  |  |  |  | SMARCB1 |
|  |  |  |  |  |  | UGT2B15 |
|  |  |  |  |  |  | CD82 |
|  |  |  |  |  |  | BCHE |
|  |  |  |  |  |  | MUC1 |
|  |  |  |  |  |  | SKP2 |
|  |  |  |  |  |  | BFIC4 |
|  |  |  |  |  |  | PART1 |
|  |  |  |  |  |  | TNFRSF10A |
|  |  |  |  |  |  | KL |
|  |  |  |  |  |  | PTHLH |
|  |  |  |  |  |  | TNFRSF11B |
|  |  |  |  |  |  | MIR27A |
|  |  |  |  |  |  | ALCAM |
|  |  |  |  |  |  | CD36 |
|  |  |  |  |  |  | ID1 |
|  |  |  |  |  |  | COL18A1 |
|  |  |  |  |  |  | MIR10A |
|  |  |  |  |  |  | CDS1 |
|  |  |  |  |  |  | HPC13 |
|  |  |  |  |  |  | TNXB |
|  |  |  |  |  |  | HPC11 |
|  |  |  |  |  |  | COPEB |
|  |  |  |  |  |  | SGP2 |
|  |  |  |  |  |  | HSD17B4 |
|  |  |  |  |  |  | IDH2 |
|  |  |  |  |  |  | MIR320A |
|  |  |  |  |  |  | NPM1 |
|  |  |  |  |  |  | DNMT3B |
|  |  |  |  |  |  | ELAC2 |
|  |  |  |  |  |  | TERC |
|  |  |  |  |  |  | EBN2 |
|  |  |  |  |  |  | EDNRA |
|  |  |  |  |  |  | CDKN2B-AS1 |
|  |  |  |  |  |  | PTENP1 |
|  |  |  |  |  |  | CXCR2 |
|  |  |  |  |  |  | GHRL |
|  |  |  |  |  |  | IRS1 |
|  |  |  |  |  |  | MIRLET7C |
|  |  |  |  |  |  | GNA11 |
|  |  |  |  |  |  | MIR181B1 |
|  |  |  |  |  |  | CLI |
|  |  |  |  |  |  | NR0B1 |
|  |  |  |  |  |  | RYR1 |
|  |  |  |  |  |  | GNRHR |
|  |  |  |  |  |  | FGF8 |
|  |  |  |  |  |  | BROVCA2 |
|  |  |  |  |  |  | OXTR |
|  |  |  |  |  |  | PRKD3 |
|  |  |  |  |  |  | APOB |
|  |  |  |  |  |  | ACTC1 |
|  |  |  |  |  |  | CDC25C |
|  |  |  |  |  |  | MIR23A |
|  |  |  |  |  |  | CYP11B2 |
|  |  |  |  |  |  | TSC2 |
|  |  |  |  |  |  | AHO |
|  |  |  |  |  |  | ETS2 |
|  |  |  |  |  |  | CDH2 |
|  |  |  |  |  |  | NCAM1 |
|  |  |  |  |  |  | EZR |
|  |  |  |  |  |  | FGFR3 |
|  |  |  |  |  |  | MIR203A |
|  |  |  |  |  |  | FLNC |
|  |  |  |  |  |  | KRAS |
|  |  |  |  |  |  | KDM4B |
|  |  |  |  |  |  | CDX2 |
|  |  |  |  |  |  | C4A |
|  |  |  |  |  |  | FGF1 |
|  |  |  |  |  |  | IL6R |
|  |  |  |  |  |  | HOXB13 |
|  |  |  |  |  |  | CDC6 |
|  |  |  |  |  |  | COL7A1 |
|  |  |  |  |  |  | JAG1 |
|  |  |  |  |  |  | CHGA |
|  |  |  |  |  |  | HSPA4 |
|  |  |  |  |  |  | PKHD1 |
|  |  |  |  |  |  | NEAT1 |
|  |  |  |  |  |  | RHOA |
|  |  |  |  |  |  | MUTYH |
|  |  |  |  |  |  | RARB |
|  |  |  |  |  |  | PRKCD |
|  |  |  |  |  |  | TP73 |
|  |  |  |  |  |  | CALCA |
|  |  |  |  |  |  | CWS1 |
|  |  |  |  |  |  | AKR1C2 |
|  |  |  |  |  |  | COL4A3 |
|  |  |  |  |  |  | PPIG |
|  |  |  |  |  |  | ADAMTS2 |
|  |  |  |  |  |  | RET |
|  |  |  |  |  |  | XPC |
|  |  |  |  |  |  | MED12 |
|  |  |  |  |  |  | HLA-DQB1 |
|  |  |  |  |  |  | STAT5A |
|  |  |  |  |  |  | DRT |
|  |  |  |  |  |  | NRAS |
|  |  |  |  |  |  | AURKA |
|  |  |  |  |  |  | SRD5A2 |
|  |  |  |  |  |  | EGR1 |
|  |  |  |  |  |  | ASAH1 |
|  |  |  |  |  |  | ADRA1A |
|  |  |  |  |  |  | CIP2A |
|  |  |  |  |  |  | CLDN7 |
|  |  |  |  |  |  | OGG1 |
|  |  |  |  |  |  | HDAC9 |
|  |  |  |  |  |  | LINC00963 |
|  |  |  |  |  |  | AFP |
|  |  |  |  |  |  | KRT15 |
|  |  |  |  |  |  | HSD3BP4 |
|  |  |  |  |  |  | MIR182 |
|  |  |  |  |  |  | PCBC |
|  |  |  |  |  |  | HPC3 |
|  |  |  |  |  |  | MTR |
|  |  |  |  |  |  | FN1 |
|  |  |  |  |  |  | PCA3 |
|  |  |  |  |  |  | ABCB1 |
|  |  |  |  |  |  | KRT17 |
|  |  |  |  |  |  | ALPP |
|  |  |  |  |  |  | KLF6 |
|  |  |  |  |  |  | NR5A1 |
|  |  |  |  |  |  | BCDS1 |
|  |  |  |  |  |  | GAA |
|  |  |  |  |  |  | IFI27 |
|  |  |  |  |  |  | HP |
|  |  |  |  |  |  | BTK |
|  |  |  |  |  |  | IL17A |
|  |  |  |  |  |  | FBI1 |
|  |  |  |  |  |  | YY1 |
|  |  |  |  |  |  | WNT3A |
|  |  |  |  |  |  | MTOR |
|  |  |  |  |  |  | ATP7B |
|  |  |  |  |  |  | CHRM4 |
|  |  |  |  |  |  | BMP6 |
|  |  |  |  |  |  | LAMB3 |
|  |  |  |  |  |  | PCAT114 |
|  |  |  |  |  |  | RTEL1 |
|  |  |  |  |  |  | PCAT1 |
|  |  |  |  |  |  | KDM1A |
|  |  |  |  |  |  | BFIC2 |
|  |  |  |  |  |  | WT1 |
|  |  |  |  |  |  | BNIP3 |
|  |  |  |  |  |  | ILK |
|  |  |  |  |  |  | SNAI1 |
|  |  |  |  |  |  | NCOA3 |
|  |  |  |  |  |  | LEF1 |
|  |  |  |  |  |  | CYP19A1 |
|  |  |  |  |  |  | KCNQ1OT1 |
|  |  |  |  |  |  | BPPV |
|  |  |  |  |  |  | SLC22A1 |
|  |  |  |  |  |  | FES |
|  |  |  |  |  |  | TXNRD2 |
|  |  |  |  |  |  | SOX9 |
|  |  |  |  |  |  | CYP3A5 |
|  |  |  |  |  |  | CDK6 |
|  |  |  |  |  |  | PDGFRB |
|  |  |  |  |  |  | KLLN |
|  |  |  |  |  |  | CTSB |
|  |  |  |  |  |  | TH |
|  |  |  |  |  |  | COL6A2 |
|  |  |  |  |  |  | PXN |
|  |  |  |  |  |  | SCHLAP1 |
|  |  |  |  |  |  | FANCA |
|  |  |  |  |  |  | INF2 |
|  |  |  |  |  |  | NGFR |
|  |  |  |  |  |  | NSD1 |
|  |  |  |  |  |  | NEDD4L |
|  |  |  |  |  |  | GNRH1 |
|  |  |  |  |  |  | CACNA1H |
|  |  |  |  |  |  | ERCC1 |
|  |  |  |  |  |  | FST |
|  |  |  |  |  |  | BLM |
|  |  |  |  |  |  | PAGE4 |
|  |  |  |  |  |  | SRD5B |
|  |  |  |  |  |  | CREBBP |
|  |  |  |  |  |  | HPC9 |
|  |  |  |  |  |  | ITGB1 |
|  |  |  |  |  |  | VEGFC |
|  |  |  |  |  |  | FANCD1 |
|  |  |  |  |  |  | TITF1 |
|  |  |  |  |  |  | PRCA1 |
|  |  |  |  |  |  | TMC8 |
|  |  |  |  |  |  | PAC1 |
|  |  |  |  |  |  | PDGFB |
|  |  |  |  |  |  | ELN |
|  |  |  |  |  |  | KLK2 |
|  |  |  |  |  |  | CALR |
|  |  |  |  |  |  | VIM |
|  |  |  |  |  |  | GLM2 |
|  |  |  |  |  |  | CARLO4 |
|  |  |  |  |  |  | DES |
|  |  |  |  |  |  | FGFR2 |
|  |  |  |  |  |  | ABCC3 |
|  |  |  |  |  |  | SOX10 |
|  |  |  |  |  |  | PCNA |
|  |  |  |  |  |  | NAB2 |
|  |  |  |  |  |  | PSMP |
|  |  |  |  |  |  | KIT |
|  |  |  |  |  |  | PRKAR1A |
|  |  |  |  |  |  | HSD3B1 |
|  |  |  |  |  |  | KRT8 |
|  |  |  |  |  |  | SHH |
|  |  |  |  |  |  | MIR99B |
|  |  |  |  |  |  | UCA1 |
|  |  |  |  |  |  | MIR210 |
|  |  |  |  |  |  | EDNRB |
|  |  |  |  |  |  | FHIT |
|  |  |  |  |  |  | JAK2 |
|  |  |  |  |  |  | MIR106B |
|  |  |  |  |  |  | TMEPAI |
|  |  |  |  |  |  | NR1H2 |
|  |  |  |  |  |  | PDPK1 |
|  |  |  |  |  |  | ETV4 |
|  |  |  |  |  |  | STAT3 |
|  |  |  |  |  |  | HBT |
|  |  |  |  |  |  | SPHK1 |
|  |  |  |  |  |  | MED25 |
|  |  |  |  |  |  | GLI1 |
|  |  |  |  |  |  | TNNT2 |
|  |  |  |  |  |  | ADAM9 |
|  |  |  |  |  |  | MAD1L1 |
|  |  |  |  |  |  | APAF1 |
|  |  |  |  |  |  | CA9 |
|  |  |  |  |  |  | XIAP |
|  |  |  |  |  |  | PCA2 |
|  |  |  |  |  |  | WNT5A |
|  |  |  |  |  |  | MYH11 |
|  |  |  |  |  |  | AGK |
|  |  |  |  |  |  | ENO2 |
|  |  |  |  |  |  | BMP7 |
|  |  |  |  |  |  | SDHD |
|  |  |  |  |  |  | NF1 |
|  |  |  |  |  |  | LRP5 |
|  |  |  |  |  |  | KLK4 |
|  |  |  |  |  |  | CBS |
|  |  |  |  |  |  | CTNNB1 |
|  |  |  |  |  |  | MIR221 |
|  |  |  |  |  |  | H19 |
|  |  |  |  |  |  | MIR183 |
|  |  |  |  |  |  | HPC6 |
|  |  |  |  |  |  | MIR141 |
|  |  |  |  |  |  | TGFBR1 |
|  |  |  |  |  |  | CTBP1-AS |
|  |  |  |  |  |  | CYP2C9 |
|  |  |  |  |  |  | DNMT3A |
|  |  |  |  |  |  | FSHR |
|  |  |  |  |  |  | VWF |
|  |  |  |  |  |  | BAD |
|  |  |  |  |  |  | PATE |
|  |  |  |  |  |  | ATBF1 |
|  |  |  |  |  |  | ITGA6 |
|  |  |  |  |  |  | CTSK |
|  |  |  |  |  |  | IGFBP2 |
|  |  |  |  |  |  | NOTCH2 |
|  |  |  |  |  |  | FLNB |
|  |  |  |  |  |  | PRKDC |
|  |  |  |  |  |  | MSR1 |
|  |  |  |  |  |  | TRPM2 |
|  |  |  |  |  |  | S100B |
|  |  |  |  |  |  | ZBTB7A |
|  |  |  |  |  |  | BMP2 |
|  |  |  |  |  |  | ATM |
|  |  |  |  |  |  | SOST |
|  |  |  |  |  |  | KCNN3 |
|  |  |  |  |  |  | SST |
|  |  |  |  |  |  | FKBP5 |
|  |  |  |  |  |  | MAPK7 |
|  |  |  |  |  |  | ICP1 |
|  |  |  |  |  |  | BMP4 |
|  |  |  |  |  |  | CDK4 |
|  |  |  |  |  |  | CLCN7 |
|  |  |  |  |  |  | AGTR1 |
|  |  |  |  |  |  | MCAM |
|  |  |  |  |  |  | DVL1 |
|  |  |  |  |  |  | MC1R |
|  |  |  |  |  |  | CALB2 |
|  |  |  |  |  |  | CPA4 |
|  |  |  |  |  |  | KCNH6 |
|  |  |  |  |  |  | CASP7 |
|  |  |  |  |  |  | HRAS |
|  |  |  |  |  |  | AIMAH2 |
|  |  |  |  |  |  | RNF43 |
|  |  |  |  |  |  | TNFRSF10C |
|  |  |  |  |  |  | CDC20 |
|  |  |  |  |  |  | MAP2K4 |
|  |  |  |  |  |  | TMC6 |
|  |  |  |  |  |  | KAT5 |
|  |  |  |  |  |  | CDKN2B |
|  |  |  |  |  |  | SMAD4 |
|  |  |  |  |  |  | PROS1 |
|  |  |  |  |  |  | FGFR4 |
|  |  |  |  |  |  | ACPP |
|  |  |  |  |  |  | XRCC1 |
|  |  |  |  |  |  | STAT6 |
|  |  |  |  |  |  | PCAT8 |
|  |  |  |  |  |  | CDH11 |
|  |  |  |  |  |  | PIK3CD |
|  |  |  |  |  |  | AREG |
|  |  |  |  |  |  | GLI3 |
|  |  |  |  |  |  | ITGA2 |
|  |  |  |  |  |  | ANO7 |
|  |  |  |  |  |  | PBOV1 |
|  |  |  |  |  |  | PTCH1 |
|  |  |  |  |  |  | MME |
|  |  |  |  |  |  | AMACR |
|  |  |  |  |  |  | S100A1 |
|  |  |  |  |  |  | PAX2 |
|  |  |  |  |  |  | DEE13 |
|  |  |  |  |  |  | GAST |
|  |  |  |  |  |  | GREM1 |
|  |  |  |  |  |  | PRKAG2 |
|  |  |  |  |  |  | SBMA |
|  |  |  |  |  |  | ADRA1D |
|  |  |  |  |  |  | NTS |
|  |  |  |  |  |  | BIRC2 |
|  |  |  |  |  |  | NTRK3 |
|  |  |  |  |  |  | SOCS3 |
|  |  |  |  |  |  | TMEFF2 |
|  |  |  |  |  |  | KIAA1888 |
|  |  |  |  |  |  | ICCA |
|  |  |  |  |  |  | FBN1 |
|  |  |  |  |  |  | WWOX |
|  |  |  |  |  |  | TIMP1 |
|  |  |  |  |  |  | DYNC2H1 |
|  |  |  |  |  |  | ARID1B |
|  |  |  |  |  |  | PKC |
|  |  |  |  |  |  | IFCR |
|  |  |  |  |  |  | CHKA |
|  |  |  |  |  |  | MGMT |
|  |  |  |  |  |  | AIP |
|  |  |  |  |  |  | NKX2-1 |
|  |  |  |  |  |  | LAMA3 |
|  |  |  |  |  |  | GPSA |
|  |  |  |  |  |  | RDH11 |
|  |  |  |  |  |  | MIR21 |
|  |  |  |  |  |  | EIF3H |
|  |  |  |  |  |  | DIS3L2 |
|  |  |  |  |  |  | EKD1 |
|  |  |  |  |  |  | FOXO1 |
|  |  |  |  |  |  | CTCF |
|  |  |  |  |  |  | MCL1 |
|  |  |  |  |  |  | EHBP1 |
|  |  |  |  |  |  | IBSP |
|  |  |  |  |  |  | MIR125A |
|  |  |  |  |  |  | PIAS1 |
|  |  |  |  |  |  | CRISP3 |
|  |  |  |  |  |  | PDE6G |
|  |  |  |  |  |  | NEB |
|  |  |  |  |  |  | KLK15 |
|  |  |  |  |  |  | TMPRSS2 |
|  |  |  |  |  |  | ANXA5 |
|  |  |  |  |  |  | CREB3L4 |
|  |  |  |  |  |  | STMN1 |
|  |  |  |  |  |  | SLC2A1 |
|  |  |  |  |  |  | EEF1A1 |
|  |  |  |  |  |  | KCNQ1 |
|  |  |  |  |  |  | RAD53 |
|  |  |  |  |  |  | EPCAM |
|  |  |  |  |  |  | HMGA2 |
|  |  |  |  |  |  | EPOR |
|  |  |  |  |  |  | NKX2A |
|  |  |  |  |  |  | TRPM4 |
|  |  |  |  |  |  | SLC22A3 |
|  |  |  |  |  |  | DHRS9 |
|  |  |  |  |  |  | EPHT3 |
|  |  |  |  |  |  | DRD1B |
|  |  |  |  |  |  | HSP90AA1 |
|  |  |  |  |  |  | EFEMP1 |
|  |  |  |  |  |  | CYP21 |
|  |  |  |  |  |  | BIN1 |
|  |  |  |  |  |  | ELOC |
|  |  |  |  |  |  | MIRLET7A1 |
|  |  |  |  |  |  | MIR335 |
|  |  |  |  |  |  | FRFB |
|  |  |  |  |  |  | SYP |
|  |  |  |  |  |  | KIF23 |
|  |  |  |  |  |  | ATS2 |
|  |  |  |  |  |  | PTPRC |
|  |  |  |  |  |  | EA9 |
|  |  |  |  |  |  | VDR |
|  |  |  |  |  |  | ACE |
|  |  |  |  |  |  | PCSEAT |
|  |  |  |  |  |  | YBX1 |
|  |  |  |  |  |  | ANXA2 |
|  |  |  |  |  |  | BCD1 |
|  |  |  |  |  |  | CDH3 |
|  |  |  |  |  |  | TPD52 |
|  |  |  |  |  |  | PRKCE |
|  |  |  |  |  |  | FH |
|  |  |  |  |  |  | PHP1B |
|  |  |  |  |  |  | CD34 |
|  |  |  |  |  |  | DEE7 |
|  |  |  |  |  |  | PDPN |
|  |  |  |  |  |  | CACNA1C |
|  |  |  |  |  |  | ENG |
|  |  |  |  |  |  | FASLG |
|  |  |  |  |  |  | MIR126 |
|  |  |  |  |  |  | CACNA1D |
|  |  |  |  |  |  | WNT1 |
|  |  |  |  |  |  | KDR |
|  |  |  |  |  |  | COL4A2 |
|  |  |  |  |  |  | KRT14 |
|  |  |  |  |  |  | ORM |
|  |  |  |  |  |  | POH |
|  |  |  |  |  |  | HNF1A |
|  |  |  |  |  |  | MIR195 |
|  |  |  |  |  |  | FLT1 |
|  |  |  |  |  |  | FIC1 |
|  |  |  |  |  |  | MEN1 |
|  |  |  |  |  |  | ALOX12 |
|  |  |  |  |  |  | DD3 |
|  |  |  |  |  |  | EXT1 |
|  |  |  |  |  |  | HOTAIR |
|  |  |  |  |  |  | EIF4E |
|  |  |  |  |  |  | SCN2A1 |
|  |  |  |  |  |  | MIR205 |
|  |  |  |  |  |  | TNC |
|  |  |  |  |  |  | SMAD2 |
|  |  |  |  |  |  | CPLANE1 |
|  |  |  |  |  |  | KMT2D |
|  |  |  |  |  |  | HPC12 |
|  |  |  |  |  |  | BGLAP |
|  |  |  |  |  |  | MIR145 |
|  |  |  |  |  |  | CCR6 |
|  |  |  |  |  |  | LHCGR |
|  |  |  |  |  |  | PFIC1 |
|  |  |  |  |  |  | F13A1 |
|  |  |  |  |  |  | GATA3 |
|  |  |  |  |  |  | BRCA2 |
|  |  |  |  |  |  | ALOX15B |
|  |  |  |  |  |  | IL2RA |
|  |  |  |  |  |  | CD24 |
|  |  |  |  |  |  | KCNJ1 |
|  |  |  |  |  |  | PNCA2 |
|  |  |  |  |  |  | SLC12A3 |
|  |  |  |  |  |  | FLCN |
|  |  |  |  |  |  | PSAP |
|  |  |  |  |  |  | BFNC2 |
|  |  |  |  |  |  | ATP1A2 |
|  |  |  |  |  |  | TG |
|  |  |  |  |  |  | ACE1 |
|  |  |  |  |  |  | MIR34A |
|  |  |  |  |  |  | BCL2L11 |
|  |  |  |  |  |  | DKBI |
|  |  |  |  |  |  | TGFB3 |
|  |  |  |  |  |  | CTNS |
|  |  |  |  |  |  | CTNNA1 |
|  |  |  |  |  |  | MIR222 |
|  |  |  |  |  |  | HGD |
|  |  |  |  |  |  | BMPR2 |
|  |  |  |  |  |  | CRH |
|  |  |  |  |  |  | AGRN |
|  |  |  |  |  |  | MAP2K5 |
|  |  |  |  |  |  | CEP290 |
|  |  |  |  |  |  | HSD17B3 |
|  |  |  |  |  |  | HPCX2 |
|  |  |  |  |  |  | WDFY3 |
|  |  |  |  |  |  | MIR214 |
|  |  |  |  |  |  | TSC1 |
|  |  |  |  |  |  | LINC01190 |
|  |  |  |  |  |  | DNMT1 |
|  |  |  |  |  |  | ID4 |
|  |  |  |  |  |  | BRIC2 |
|  |  |  |  |  |  | PRKG1 |
|  |  |  |  |  |  | FTO |
|  |  |  |  |  |  | CYP27B1 |
|  |  |  |  |  |  | SLC6A1 |
|  |  |  |  |  |  | PLA2G5 |
|  |  |  |  |  |  | PALB2 |
|  |  |  |  |  |  | ZF9 |
|  |  |  |  |  |  | IGF2-AS |
|  |  |  |  |  |  | MMP14 |
|  |  |  |  |  |  | PCAT2 |
|  |  |  |  |  |  | MDM2 |
|  |  |  |  |  |  | COL4A4 |
|  |  |  |  |  |  | FGF7 |
|  |  |  |  |  |  | TNFSF11 |
|  |  |  |  |  |  | SPOP |
|  |  |  |  |  |  | ADH |
|  |  |  |  |  |  | AKT2 |
|  |  |  |  |  |  | LIFR |
|  |  |  |  |  |  | CYP3A7 |
|  |  |  |  |  |  | CBL |
|  |  |  |  |  |  | NDRG1 |
|  |  |  |  |  |  | SP1 |
|  |  |  |  |  |  | THBS4 |
|  |  |  |  |  |  | KD |
|  |  |  |  |  |  | AXIN2 |
|  |  |  |  |  |  | MYLK |
|  |  |  |  |  |  | MXI1 |
|  |  |  |  |  |  | MIR23B |
|  |  |  |  |  |  | MIR200A |
|  |  |  |  |  |  | PIK3CA |
|  |  |  |  |  |  | LOC111099028 |
|  |  |  |  |  |  | CHD2 |
|  |  |  |  |  |  | MIRLET7B |
|  |  |  |  |  |  | CTLA4 |
|  |  |  |  |  |  | BFIS3 |
|  |  |  |  |  |  | PCGEM1 |
|  |  |  |  |  |  | DAB2IP |
|  |  |  |  |  |  | KDM4C |
|  |  |  |  |  |  | FADD |
|  |  |  |  |  |  | FOXA1 |
|  |  |  |  |  |  | MCM7 |
|  |  |  |  |  |  | LYVE1 |
|  |  |  |  |  |  | CREB1 |
|  |  |  |  |  |  | BFIS2 |
|  |  |  |  |  |  | SYK |
|  |  |  |  |  |  | IGFBP1 |
|  |  |  |  |  |  | CEACAM5 |
|  |  |  |  |  |  | SRD5A1 |
|  |  |  |  |  |  | LGALS3 |
|  |  |  |  |  |  | CYS1 |
|  |  |  |  |  |  | CDH1 |
|  |  |  |  |  |  | PSAT1 |
|  |  |  |  |  |  | PLAG1 |
|  |  |  |  |  |  | PCAT18 |
|  |  |  |  |  |  | SCN2A |
|  |  |  |  |  |  | IL4 |
|  |  |  |  |  |  | DIABLO |
|  |  |  |  |  |  | SMARCA4 |
|  |  |  |  |  |  | SLC18A2 |
|  |  |  |  |  |  | PRG2 |
|  |  |  |  |  |  | GNAQ |
|  |  |  |  |  |  | KCNQ2 |
|  |  |  |  |  |  | MIR142 |
|  |  |  |  |  |  | CCNG1 |
|  |  |  |  |  |  | KRT20 |
|  |  |  |  |  |  | BFIS1 |
|  |  |  |  |  |  | SPDEF |
|  |  |  |  |  |  | FGF2 |
|  |  |  |  |  |  | AZGP1 |
|  |  |  |  |  |  | CLU |
|  |  |  |  |  |  | STAT5B |
|  |  |  |  |  |  | AMH |
|  |  |  |  |  |  | HSD3B2 |
|  |  |  |  |  |  | CXCL12 |
|  |  |  |  |  |  | WDR77 |
|  |  |  |  |  |  | TET2 |
|  |  |  |  |  |  | COL2A1 |
|  |  |  |  |  |  | PCAT4 |
|  |  |  |  |  |  | MIR224 |
|  |  |  |  |  |  | GLUL |
|  |  |  |  |  |  | MRE11 |
|  |  |  |  |  |  | KLF5 |
|  |  |  |  |  |  | LGI1 |
|  |  |  |  |  |  | PDE5A |
|  |  |  |  |  |  | BFIC3 |
|  |  |  |  |  |  | IGF1R |
|  |  |  |  |  |  | COL14A1 |
|  |  |  |  |  |  | PRKD1 |
|  |  |  |  |  |  | MIR199B |
|  |  |  |  |  |  | HPC2 |
|  |  |  |  |  |  | RECK |
|  |  |  |  |  |  | STAR |
|  |  |  |  |  |  | F2R |
|  |  |  |  |  |  | RTEL1-TNFRSF6B |
|  |  |  |  |  |  | MIR191 |
|  |  |  |  |  |  | THBS1 |
|  |  |  |  |  |  | ST12 |
|  |  |  |  |  |  | CHK2 |
|  |  |  |  |  |  | TSG101 |
|  |  |  |  |  |  | CD19 |
|  |  |  |  |  |  | PCAP |
|  |  |  |  |  |  | RNASEL |
|  |  |  |  |  |  | GNAS1 |
|  |  |  |  |  |  | MAPK3 |
|  |  |  |  |  |  | NCOR1 |
|  |  |  |  |  |  | MIR143 |
|  |  |  |  |  |  | SPINT1 |
|  |  |  |  |  |  | TTF1 |
|  |  |  |  |  |  | VIP |
|  |  |  |  |  |  | ICH |
|  |  |  |  |  |  | MC2R |
|  |  |  |  |  |  | HDAC2 |
|  |  |  |  |  |  | PLEC |
|  |  |  |  |  |  | LRP2 |
|  |  |  |  |  |  | IL6ST |
|  |  |  |  |  |  | GPER1 |
|  |  |  |  |  |  | ETS1 |
|  |  |  |  |  |  | HTGH |
|  |  |  |  |  |  | ERK |
|  |  |  |  |  |  | RAD50 |
|  |  |  |  |  |  | MYCN |
|  |  |  |  |  |  | MIR181A1 |
|  |  |  |  |  |  | HPC5 |
|  |  |  |  |  |  | EIF4EBP1 |
|  |  |  |  |  |  | ABCB4 |
|  |  |  |  |  |  | CXCL1 |
|  |  |  |  |  |  | PC |
|  |  |  |  |  |  | BFIS5 |
|  |  |  |  |  |  | STEAP2 |
|  |  |  |  |  |  | BRIC |
|  |  |  |  |  |  | HNF4A |
|  |  |  |  |  |  | MYH7 |
|  |  |  |  |  |  | TCIRG1 |
|  |  |  |  |  |  | TNFRSF10B |
|  |  |  |  |  |  | TERT |
|  |  |  |  |  |  | MIR503 |
|  |  |  |  |  |  | MIR106A |
|  |  |  |  |  |  | MCM2 |
|  |  |  |  |  |  | PAX8 |
|  |  |  |  |  |  | KRT18 |
|  |  |  |  |  |  | GRPR |
|  |  |  |  |  |  | HPC10 |
|  |  |  |  |  |  | TGFA |
|  |  |  |  |  |  | TNFRSF11A |
|  |  |  |  |  |  | PTH |
|  |  |  |  |  |  | PROM1 |
|  |  |  |  |  |  | MIR10B |
|  |  |  |  |  |  | PRLR |
|  |  |  |  |  |  | ZFYVE26 |
|  |  |  |  |  |  | CYP11B1 |
|  |  |  |  |  |  | CDC73 |
|  |  |  |  |  |  | CFTR |
|  |  |  |  |  |  | MIR96 |
|  |  |  |  |  |  | BAP1 |
|  |  |  |  |  |  | MIR25 |
|  |  |  |  |  |  | MSH2 |
|  |  |  |  |  |  | ST14 |
|  |  |  |  |  |  | CYLD |
|  |  |  |  |  |  | HBB |
|  |  |  |  |  |  | SRC |
|  |  |  |  |  |  | CASR |
|  |  |  |  |  |  | CDK2 |
|  |  |  |  |  |  | EBN1 |
|  |  |  |  |  |  | BUB1B |
|  |  |  |  |  |  | MIR16-1 |
|  |  |  |  |  |  | MT-CO1 |
|  |  |  |  |  |  | CCNH |
|  |  |  |  |  |  | DKK1 |
|  |  |  |  |  |  | BFIC1 |
|  |  |  |  |  |  | SLC12A5 |
|  |  |  |  |  |  | IL1RN |
|  |  |  |  |  |  | ERCC6 |
|  |  |  |  |  |  | KRT7 |
|  |  |  |  |  |  | MIR296 |
|  |  |  |  |  |  | SSTR1 |
|  |  |  |  |  |  | MIR139 |
|  |  |  |  |  |  | PDE11A |
|  |  |  |  |  |  | PSM |
|  |  |  |  |  |  | CDKN1C |
|  |  |  |  |  |  | POLE |
|  |  |  |  |  |  | CIAT |
|  |  |  |  |  |  | COL17A1 |
|  |  |  |  |  |  | GFAP |
|  |  |  |  |  |  | CAPB |
|  |  |  |  |  |  | CYP17A1 |
|  |  |  |  |  |  | MIR22 |
|  |  |  |  |  |  | DRD1L2 |
|  |  |  |  |  |  | LINC01092 |
|  |  |  |  |  |  | KRT19 |
|  |  |  |  |  |  | ADRA1 |
|  |  |  |  |  |  | SDHC |
|  |  |  |  |  |  | VCL |
|  |  |  |  |  |  | HTC3 |
|  |  |  |  |  |  | LRF |
|  |  |  |  |  |  | ECAD |
|  |  |  |  |  |  | HFE |
|  |  |  |  |  |  | COL4A1 |
|  |  |  |  |  |  | SUFU |
|  |  |  |  |  |  | VCAN |
|  |  |  |  |  |  | FOLH |
|  |  |  |  |  |  | BSEP |
|  |  |  |  |  |  | MKI67 |
|  |  |  |  |  |  | DNAH8 |
|  |  |  |  |  |  | MIR17 |
|  |  |  |  |  |  | GLI2 |
|  |  |  |  |  |  | BTNL2 |
|  |  |  |  |  |  | CCL5 |
|  |  |  |  |  |  | TGIF1 |
|  |  |  |  |  |  | EWSR1 |
|  |  |  |  |  |  | CR2 |
|  |  |  |  |  |  | OR51E2 |
|  |  |  |  |  |  | JUP |
|  |  |  |  |  |  | STK11 |
|  |  |  |  |  |  | TGFBR2 |
|  |  |  |  |  |  | CDH23 |
|  |  |  |  |  |  | TTN |
|  |  |  |  |  |  | KIAA0903 |
|  |  |  |  |  |  | LOX |
|  |  |  |  |  |  | RELB |
|  |  |  |  |  |  | HPC7 |
|  |  |  |  |  |  | HNF1B |
|  |  |  |  |  |  | GDEP |
|  |  |  |  |  |  | CCN1 |
|  |  |  |  |  |  | SLC45A3 |
|  |  |  |  |  |  | HLA-DRB1 |
|  |  |  |  |  |  | MEG3 |
|  |  |  |  |  |  | RAD51 |
|  |  |  |  |  |  | SRA1 |
|  |  |  |  |  |  | IER3IP1 |
|  |  |  |  |  |  | LOC111099027 |
|  |  |  |  |  |  | KLK10 |
|  |  |  |  |  |  | NOG |
|  |  |  |  |  |  | MLANA |
|  |  |  |  |  |  | LAMC2 |
|  |  |  |  |  |  | TGM4 |
|  |  |  |  |  |  | ADAM17 |
|  |  |  |  |  |  | JAK1 |
|  |  |  |  |  |  | CTNND1 |
|  |  |  |  |  |  | HOXB9 |
|  |  |  |  |  |  | TXBP181 |
|  |  |  |  |  |  | RARRES1 |
|  |  |  |  |  |  | G6PC1 |
|  |  |  |  |  |  | DEE11 |
|  |  |  |  |  |  | UC28 |
|  |  |  |  |  |  | UROC28 |
|  |  |  |  |  |  | CCND2 |
|  |  |  |  |  |  | CFLAR |
|  |  |  |  |  |  | MYOCL2 |
|  |  |  |  |  |  | PIK3R1 |
|  |  |  |  |  |  | GPC3 |
|  |  |  |  |  |  | GRP |
|  |  |  |  |  |  | MAF |
|  |  |  |  |  |  | PDGFRA |
|  |  |  |  |  |  | STXBP1 |
|  |  |  |  |  |  | CSTB |
|  |  |  |  |  |  | DMD |
|  |  |  |  |  |  | COL6A3 |
|  |  |  |  |  |  | DLC1 |
|  |  |  |  |  |  | BFNS1 |
|  |  |  |  |  |  | PRKACA |
|  |  |  |  |  |  | MIR199A1 |
|  |  |  |  |  |  | KCNQ3 |
|  |  |  |  |  |  | SCN8A |
|  |  |  |  |  |  | ABCB11 |
|  |  |  |  |  |  | ZFHX3 |
|  |  |  |  |  |  | ASXL1 |
|  |  |  |  |  |  | KCNH7 |
|  |  |  |  |  |  | CIPAR1 |
|  |  |  |  |  |  | UVO |
|  |  |  |  |  |  | HADHA |
|  |  |  |  |  |  | IDH1 |
|  |  |  |  |  |  | POU5F1 |
|  |  |  |  |  |  | INHA |
|  |  |  |  |  |  | YAP1 |
|  |  |  |  |  |  | MUC5AC |
|  |  |  |  |  |  | HDAC1 |
|  |  |  |  |  |  | BMI1 |
|  |  |  |  |  |  | MALAT1 |
|  |  |  |  |  |  | MIF |
|  |  |  |  |  |  | GH1 |
|  |  |  |  |  |  | MAP2K1 |
|  |  |  |  |  |  | NFKB1 |
|  |  |  |  |  |  | SIRT1 |
|  |  |  |  |  |  | PRAC1 |
|  |  |  |  |  |  | KRT5 |
|  |  |  |  |  |  | MIR27B |
|  |  |  |  |  |  | TIMP2 |
|  |  |  |  |  |  | LOC107133510 |
|  |  |  |  |  |  | ITGB3 |
|  |  |  |  |  |  | BDPLT22 |
|  |  |  |  |  |  | BMPR1B |
|  |  |  |  |  |  | REN |
|  |  |  |  |  |  | ACTA1 |
|  |  |  |  |  |  | PDE8B |
|  |  |  |  |  |  | BMX |
|  |  |  |  |  |  | CALD1 |
|  |  |  |  |  |  | COL6A1 |
|  |  |  |  |  |  | POMC |
|  |  |  |  |  |  | PMS1 |
|  |  |  |  |  |  | CBR3-AS1 |
|  |  |  |  |  |  | KITLG |
|  |  |  |  |  |  | ATS3 |
|  |  |  |  |  |  | VIPR1 |
|  |  |  |  |  |  | SPARC |
|  |  |  |  |  |  | P450C11 |
|  |  |  |  |  |  | COL5A1 |
|  |  |  |  |  |  | ACTA2 |
|  |  |  |  |  |  | ALB |
|  |  |  |  |  |  | LGALS8 |
|  |  |  |  |  |  | SREBF1 |
|  |  |  |  |  |  | PMEPA1 |
|  |  |  |  |  |  | ARMC5 |
|  |  |  |  |  |  | EPAS1 |
|  |  |  |  |  |  | PRNCR1 |
|  |  |  |  |  |  | MLH1 |
|  |  |  |  |  |  | KCNJ5 |
|  |  |  |  |  |  | PHP1A |
|  |  |  |  |  |  | LMNA |
|  |  |  |  |  |  | CYB5A |
|  |  |  |  |  |  | FOXO3 |
|  |  |  |  |  |  | TEPP |
|  |  |  |  |  |  | GAPDH |
|  |  |  |  |  |  | APC |
|  |  |  |  |  |  | FHI |
|  |  |  |  |  |  | PVT1 |
|  |  |  |  |  |  | GPX1 |
|  |  |  |  |  |  | WNK1 |
|  |  |  |  |  |  | CDKN2A |
|  |  |  |  |  |  | KLK11 |
|  |  |  |  |  |  | ABCA5 |
|  |  |  |  |  |  | LIMK1 |
|  |  |  |  |  |  | KCNJ11 |
|  |  |  |  |  |  | PSCA |
|  |  |  |  |  |  | RECQL4 |
|  |  |  |  |  |  | ABCC8 |
|  |  |  |  |  |  | TRPS1 |
|  |  |  |  |  |  | SERPINA3 |
|  |  |  |  |  |  | ALKBH3 |
|  |  |  |  |  |  | TSHR |
|  |  |  |  |  |  | RANBP2 |
|  |  |  |  |  |  | HPC14 |
|  |  |  |  |  |  | CSF1R |
|  |  |  |  |  |  | ETV1 |
|  |  |  |  |  |  | MMAC1 |
|  |  |  |  |  |  | PTH1R |
|  |  |  |  |  |  | NOTCH3 |
|  |  |  |  |  |  | NF2 |
|  |  |  |  |  |  | PRRT2 |
|  |  |  |  |  |  | NMTC1 |
|  |  |  |  |  |  | COXPD17 |
|  |  |  |  |  |  | EPHB2 |
|  |  |  |  |  |  | SNAI2 |
|  |  |  |  |  |  | PLAUR |
|  |  |  |  |  |  | FLT4 |
|  |  |  |  |  |  | CDKN3 |
|  |  |  |  |  |  | NME1 |
|  |  |  |  |  |  | SCAP |
|  |  |  |  |  |  | HPCQTL19 |
|  |  |  |  |  |  | MIR483 |
|  |  |  |  |  |  | E2F3 |
|  |  |  |  |  |  | PHP1C |
|  |  |  |  |  |  | CSNK2A1 |
|  |  |  |  |  |  | HSPG2 |
|  |  |  |  |  |  | BRAF |
|  |  |  |  |  |  | FLNA |
|  |  |  |  |  |  | TWIST1 |
|  |  |  |  |  |  | EPO |
|  |  |  |  |  |  | NOTCH1 |
|  |  |  |  |  |  | PEBP1 |
|  |  |  |  |  |  | CD274 |
|  |  |  |  |  |  | CP |
|  |  |  |  |  |  | AKT3 |
|  |  |  |  |  |  | SFRP1 |
|  |  |  |  |  |  | MYAS1 |
|  |  |  |  |  |  | NCOA4 |
|  |  |  |  |  |  | KLK3 |
|  |  |  |  |  |  | CDH5 |
|  |  |  |  |  |  | MNDLFH |
|  |  |  |  |  |  | CYP24A1 |
|  |  |  |  |  |  | TNFRSF10D |
|  |  |  |  |  |  | GSTT1 |
|  |  |  |  |  |  | USP8 |
|  |  |  |  |  |  | HGF |
|  |  |  |  |  |  | EPHB4 |
|  |  |  |  |  |  | TYMP |
|  |  |  |  |  |  | ATP8B1 |
|  |  |  |  |  |  | MIR29A |
|  |  |  |  |  |  | ARID1A |
|  |  |  |  |  |  | PSMA |
|  |  |  |  |  |  | SCN9A |
|  |  |  |  |  |  | SLC5A8 |
|  |  |  |  |  |  | SOX2 |
|  |  |  |  |  |  | TP63 |
|  |  |  |  |  |  | KAT2B |
|  |  |  |  |  |  | SDHA |
|  |  |  |  |  |  | GATA4 |
|  |  |  |  |  |  | GLM3 |
|  |  |  |  |  |  | ALPL |
|  |  |  |  |  |  | COMT |
|  |  |  |  |  |  | VHL |
|  |  |  |  |  |  | PARM1 |
|  |  |  |  |  |  | SAT1 |
|  |  |  |  |  |  | LINC00913 |
|  |  |  |  |  |  | MIR212 |
|  |  |  |  |  |  | FGF23 |
|  |  |  |  |  |  | PTCH2 |
|  |  |  |  |  |  | ERCC2 |
|  |  |  |  |  |  | CEACAM3 |
|  |  |  |  |  |  | HYAL1 |
|  |  |  |  |  |  | VPS13B |
|  |  |  |  |  |  | MIR375 |
|  |  |  |  |  |  | FANCD2 |
|  |  |  |  |  |  | HPC1 |
|  |  |  |  |  |  | CSF2 |
|  |  |  |  |  |  | PMS2 |
|  |  |  |  |  |  | GHRH |
|  |  |  |  |  |  | RPS6KB1 |
|  |  |  |  |  |  | BIRC3 |
|  |  |  |  |  |  | LBR |
|  |  |  |  |  |  | STEAP1 |
|  |  |  |  |  |  | SRY |
|  |  |  |  |  |  | CDC25A |
|  |  |  |  |  |  | ADIPOQ |
