# Supplementary material for: Exploring the mechanism of action of the combination of cinnamon and motherwort in the treatment of benign prostatic hyperplasia: A network pharmacology study
Source: Medicine (Baltimore). 2024 Apr 26;103(17):e37902. doi: 10.1097/MD.0000000000037902 (PMC11049697; doi:10.1097/MD.0000000000037902)
Supplement: Supplementary file 2 [file medi-103-e37902-s002.docx]

| GP-BP |  |  |  |  |  |
| --- | --- | --- | --- | --- | --- |
| Description | LogP | Enrichment | Log(q-value) | BestLogPInGroup | BestEnrichmentInGroup |
| response to hormone | -45.1954192 | 15.424456 | -41.00772927 | -45.19542 | 18.27873897 |
| cellular response to lipid | -40.1452716 | 18.9343631 | -36.25861166 | -40.14527 | 22.06776923 |
| cellular response to organic cyclic compound | -38.6111838 | 18.3993907 | -34.90061519 | -38.61118 | 18.39939071 |
| response to inorganic substance | -38.0681649 | 17.8343422 | -34.48253497 | -38.06816 | 69.05987526 |
| cellular response to organonitrogen compound | -37.60801 | 16.4206631 | -34.11929007 | -45.19542 | 18.27873897 |
| cellular response to nitrogen compound | -37.2379525 | 15.2441827 | -33.8284139 | -45.19542 | 18.27873897 |
| positive regulation of cell migration | -36.816686 | 16.5923077 | -33.47409414 | -36.81669 | 30.97230769 |
| positive regulation of cell motility | -36.0925192 | 15.910432 | -32.80791926 | -36.81669 | 30.97230769 |
| positive regulation of cellular component movement | -35.713579 | 15.5639737 | -32.48013162 | -36.81669 | 30.97230769 |
| positive regulation of locomotion | -35.6560735 | 15.5120072 | -32.46838361 | -36.81669 | 30.97230769 |
| GO-CC |  |  |  |  |  |
| Description | LogP | Enrichment | Log(q-value) | BestLogPInGroup | BestEnrichmentInGroup |
| membrane raft | -14.3708214 | 12.8259556 | -11.35943575 | -14.37082 | 22.66266417 |
| membrane microdomain | -14.3477717 | 12.7867325 | -11.35943575 | -14.37082 | 22.66266417 |
| transcription regulator complex | -10.2978015 | 7.97771562 | -7.485556819 | -10.2978 | 10.55874126 |
| vesicle lumen | -9.78712751 | 9.94523641 | -7.099821554 | -9.787128 | 20.80229621 |
| secretory granule lumen | -8.81015816 | 9.37826087 | -6.250302954 | -9.787128 | 20.80229621 |
| cytoplasmic vesicle lumen | -8.76151765 | 9.29169231 | -6.250302954 | -9.787128 | 20.80229621 |
| caveola | -8.58689411 | 22.6626642 | -6.142626194 | -14.37082 | 22.66266417 |
| RNA polymerase II transcription regulator complex | -8.05718631 | 10.5587413 | -5.670910341 | -10.2978 | 10.55874126 |
| plasma membrane raft | -7.47819361 | 16.4454731 | -5.143070165 | -14.37082 | 22.66266417 |
| protein kinase complex | -7.21768781 | 15.2322825 | -4.928321854 | -7.217688 | 23.7032967 |
| GO-MF |  |  |  |  |  |
| Description | LogP | Enrichment | Log(q-value) | BestLogPInGroup | BestEnrichmentInGroup |
| kinase binding | -21.8572388 | 9.64118527 | -18.17221397 | -21.85724 | 9.777179859 |
| protein kinase binding | -19.9127388 | 9.77717986 | -16.52874403 | -21.85724 | 9.777179859 |
| DNA-binding transcription factor binding | -19.5404163 | 12.3559738 | -16.3325128 | -19.54042 | 34.84384615 |
| transcription factor binding | -19.344368 | 10.5409955 | -16.26140324 | -19.54042 | 34.84384615 |
| protein homodimerization activity | -17.7731612 | 9.14270016 | -14.78710642 | -17.77316 | 9.142700157 |
| RNA polymerase II-specific DNA-binding transcription factor binding | -17.5203511 | 14.0176393 | -14.61347752 | -19.54042 | 34.84384615 |
| protein domain specific binding | -13.628047 | 7.77688949 | -10.78812027 | -13.62805 | 12.45731619 |
| protease binding | -13.5347948 | 22.2044118 | -10.75286 | -13.53479 | 22.20441176 |
| ubiquitin-like protein ligase binding | -13.3811279 | 12.4573162 | -10.65034566 | -13.62805 | 12.45731619 |
| protein kinase activity | -13.3254525 | 8.63387338 | -10.64042769 | -13.32545 | 8.633873383 |
