# Supplementary material for: Exploring the mechanism of action of the combination of cinnamon and motherwort in the treatment of benign prostatic hyperplasia: A network pharmacology study
Source: Medicine (Baltimore). 2024 Apr 26;103(17):e37902. doi: 10.1097/MD.0000000000037902 (PMC11049697; doi:10.1097/MD.0000000000037902)
Supplement: Supplementary file 3 [file medi-103-e37902-s003.docx]

| GO | Description | LogP | Enrichment | Log(q-value) |
| --- | --- | --- | --- | --- |
| hsa05200 | Pathways in cancer | -69.4318 | 26.24771838 | -66.89273672 |
| hsa04933 | AGE-RAGE signaling pathway in diabetic complications | -47.1822 | 69.68769231 | -44.94418751 |
| hsa05418 | Fluid shear stress and atherosclerosis | -44.2357 | 51.80619812 | -42.17371346 |
| hsa05161 | Hepatitis B | -43.8354 | 45.88490028 | -41.89836852 |
| hsa05417 | Lipid and atherosclerosis | -43.1053 | 36.7345975 | -41.26519774 |
| hsa05215 | Prostate cancer | -39.3316 | 62.26391753 | -37.57069233 |
| hsa04151 | PI3K-Akt signaling pathway | -38.5238 | 23.62294654 | -36.82980538 |
| hsa05167 | Kaposi sarcoma-associated herpesvirus infection | -37.5987 | 35.92149088 | -35.96269855 |
| hsa05163 | Human cytomegalovirus infection | -33.9061 | 29.93989744 | -32.32127446 |
| hsa04010 | MAPK signaling pathway | -33.5053 | 24.49340659 | -31.96617564 |
| hsa05142 | Chagas disease | -32.812 | 52.37963801 | -31.31427124 |
| hsa05212 | Pancreatic cancer | -32.0445 | 64.18603239 | -30.58461 |
| hsa05205 | Proteoglycans in cancer | -31.7925 | 30.59459662 | -30.38336761 |
| hsa04657 | IL-17 signaling pathway | -31.7763 | 54.36628478 | -30.38336761 |
| hsa05162 | Measles | -31.2089 | 40.10802435 | -29.84595436 |
| hsa05166 | Human T-cell leukemia virus 1 infection | -30.8206 | 28.25176715 | -29.48566171 |
| hsa05164 | Influenza A | -30.5983 | 33.9608637 | -29.28964492 |
| hsa05145 | Toxoplasmosis | -29.929 | 45.62884615 | -28.64523127 |
| hsa05207 | Chemical carcinogenesis - receptor activation | -29.7612 | 28.48867925 | -28.50088239 |
| hsa05219 | Bladder cancer | -29.5518 | 96.3163227 | -28.31374663 |
| hsa05225 | Hepatocellular carcinoma | -29.1103 | 33.18461538 | -27.89348554 |
| hsa05169 | Epstein-Barr virus infection | -28.7063 | 28.74904798 | -27.50967013 |
| hsa04066 | HIF-1 signaling pathway | -28.3746 | 44.75356387 | -27.19726455 |
| hsa04218 | Cellular senescence | -28.2223 | 34.24822485 | -27.06343371 |
| hsa05160 | Hepatitis C | -28.155 | 34.03008329 | -27.01385105 |
| hsa04668 | TNF signaling pathway | -28.1059 | 43.55480769 | -26.98176 |
| hsa04210 | Apoptosis | -27.9305 | 37.57669683 | -26.8227549 |
| hsa01522 | Endocrine resistance | -27.5707 | 47.40659341 | -26.47881166 |
| hsa05210 | Colorectal cancer | -26.9173 | 51.32039356 | -25.84066372 |
| hsa04926 | Relaxin signaling pathway | -26.7213 | 37.81502683 | -25.6592993 |
| hsa05222 | Small cell lung cancer | -26.3044 | 47.97341137 | -25.2566688 |
| hsa05208 | Chemical carcinogenesis - reactive oxygen species | -26.0477 | 25.00006899 | -25.01374157 |
| hsa05224 | Breast cancer | -25.4608 | 33.18461538 | -24.44021198 |
| hsa05152 | Tuberculosis | -25.1191 | 28.39128205 | -24.11151706 |
| hsa05165 | Human papillomavirus infection | -24.647 | 18.24652568 | -23.65199213 |
| hsa05135 | Yersinia infection | -24.4488 | 33.91128579 | -23.46604491 |
| hsa05140 | Leishmaniasis | -24.0972 | 51.28531469 | -23.12633027 |
| hsa04620 | Toll-like receptor signaling pathway | -23.4317 | 40.20443787 | -22.48367014 |
| hsa04625 | C-type lectin receptor signaling pathway | -23.4317 | 40.20443787 | -22.48367014 |
| hsa05132 | Salmonella infection | -23.404 | 21.45671918 | -22.46700996 |
| hsa04932 | Non-alcoholic fatty liver disease | -23.327 | 29.97320099 | -22.40066685 |
| hsa05235 | PD-L1 expression and PD-1 checkpoint pathway in cancer | -22.9267 | 44.3704408 | -22.01090721 |
| hsa05213 | Endometrial cancer | -22.4469 | 60.07559682 | -21.54132462 |
| hsa05220 | Chronic myeloid leukemia | -22.3309 | 48.90364372 | -21.43526844 |
| hsa05170 | Human immunodeficiency virus 1 infection | -22.0126 | 23.01008708 | -21.12671519 |
| hsa04068 | FoxO signaling pathway | -21.5221 | 31.91802701 | -20.6458167 |
| hsa05206 | MicroRNAs in cancer | -21.2142 | 17.23459057 | -20.34723315 |
| hsa05171 | Coronavirus disease - COVID-19 | -21.181 | 21.02645889 | -20.32315038 |
| hsa04510 | Focal adhesion | -21.0077 | 23.11366246 | -20.15880392 |
| hsa05223 | Non-small cell lung cancer | -20.8795 | 48.39423077 | -20.03937396 |
| hsa01524 | Platinum drug resistance | -20.7811 | 47.7312961 | -19.95806799 |
| hsa04115 | p53 signaling pathway | -20.7811 | 47.7312961 | -19.95806799 |
| hsa05133 | Pertussis | -20.495 | 45.84716599 | -19.68023389 |
| hsa01521 | EGFR tyrosine kinase inhibitor resistance | -20.2214 | 44.10613437 | -19.41471866 |
| hsa04380 | Osteoclast differentiation | -20.0776 | 30.85132212 | -19.2789075 |
| hsa04660 | T cell receptor signaling pathway | -19.985 | 35.73727811 | -19.194127 |
| hsa04014 | Ras signaling pathway | -19.7505 | 20.02519894 | -18.96727011 |
| hsa04659 | Th17 cell differentiation | -19.7084 | 34.41367521 | -18.93517837 |
| hsa05130 | Pathogenic Escherichia coli infection | -19.7034 | 22.40382663 | -18.93517837 |
| hsa05022 | Pathways of neurodegeneration - multiple diseases | -19.4079 | 12.20022624 | -18.6469834 |
| hsa04936 | Alcoholic liver disease | -19.2833 | 27.80964247 | -18.52954154 |
| hsa05131 | Shigellosis | -19.2062 | 18.80909374 | -18.45952851 |
| hsa05323 | Rheumatoid arthritis | -19.0822 | 37.46650124 | -18.34243181 |
| hsa04621 | NOD-like receptor signaling pathway | -18.7893 | 22.72424749 | -18.05640548 |
| hsa05146 | Amoebiasis | -18.4463 | 34.16063348 | -17.72014327 |
| hsa05415 | Diabetic cardiomyopathy | -18.0143 | 20.59734748 | -17.29473976 |
| hsa04012 | ErbB signaling pathway | -17.9694 | 38.2599095 | -17.25641556 |
| hsa04915 | Estrogen signaling pathway | -17.9368 | 26.93244147 | -17.23020023 |
| hsa05010 | Alzheimer disease | -17.8709 | 13.30841346 | -17.17070783 |
| hsa04917 | Prolactin signaling pathway | -17.4396 | 43.14 | -16.7456153 |
| hsa04722 | Neurotrophin signaling pathway | -17.3985 | 29.28054299 | -16.71063738 |
| hsa05226 | Gastric cancer | -17.3908 | 24.94414042 | -16.70909293 |
| hsa04919 | Thyroid hormone signaling pathway | -17.2861 | 28.79656707 | -16.61034403 |
| hsa05214 | Glioma | -17.0214 | 40.264 | -16.35153042 |
| hsa04630 | JAK-STAT signaling pathway | -16.7996 | 22.94245014 | -16.13563165 |
| hsa04064 | NF-kappa B signaling pathway | -16.6784 | 31.27011834 | -16.02011956 |
| hsa05321 | Inflammatory bowel disease | -16.0969 | 42.88473373 | -15.44433334 |
| hsa04071 | Sphingolipid signaling pathway | -15.8305 | 27.32850679 | -15.18347478 |
| hsa05144 | Malaria | -15.7035 | 51.10430769 | -15.06202706 |
| hsa05218 | Melanoma | -15.5265 | 38.71538462 | -14.89051263 |
| hsa05202 | Transcriptional misregulation in cancer | -14.2062 | 18.0538063 | -13.57561565 |
| hsa04658 | Th1 and Th2 cell differentiation | -14.1847 | 30.29899666 | -13.55943829 |
| hsa04931 | Insulin resistance | -13.3241 | 25.81025641 | -12.70415021 |
| hsa05134 | Legionellosis | -13.2524 | 40.75303644 | -12.63762142 |
| hsa04370 | VEGF signaling pathway | -13.0927 | 39.37157757 | -12.48306284 |
| hsa04921 | Oxytocin signaling pathway | -12.8304 | 19.60909091 | -12.22577427 |
| hsa05221 | Acute myeloid leukemia | -12.509 | 34.67049369 | -11.9138625 |
| hsa04110 | Cell cycle | -12.5085 | 22.12307692 | -11.9138625 |
| hsa05120 | Epithelial cell signaling in Helicobacter pylori infection | -12.3097 | 33.18461538 | -11.72486949 |
| hsa05230 | Central carbon metabolism in cancer | -12.3097 | 33.18461538 | -11.72486949 |
| hsa04215 | Apoptosis - multiple species | -12.0388 | 58.07307692 | -11.45878941 |
| hsa04662 | B cell receptor signaling pathway | -11.5968 | 28.32833021 | -11.02148082 |
| hsa04020 | Calcium signaling pathway | -11.5768 | 13.55038462 | -11.00625659 |
| hsa05168 | Herpes simplex virus 1 infection | -11.2947 | 8.446993007 | -10.72879981 |
| hsa05203 | Viral carcinogenesis | -11.2648 | 14.80294118 | -10.70340538 |
| hsa04024 | cAMP signaling pathway | -10.8262 | 13.66425339 | -10.27226437 |
| hsa05020 | Prion disease | -10.8246 | 11.91242604 | -10.27226437 |
| hsa05231 | Choline metabolism in cancer | -10.8059 | 23.7032967 | -10.25803954 |
| hsa04622 | RIG-I-like receptor signaling pathway | -10.6994 | 29.86615385 | -10.15591026 |
| hsa01523 | Antifolate resistance | -10.3602 | 54.20153846 | -9.821145793 |
| hsa04062 | Chemokine signaling pathway | -10.3347 | 14.51826923 | -9.799895344 |
| hsa04150 | mTOR signaling pathway | -10.076 | 16.3795858 | -9.545513204 |
| hsa04217 | Necroptosis | -9.98669 | 16.07053701 | -9.460447753 |
| hsa04935 | Growth hormone synthesis, secretion and action | -9.95828 | 19.52036199 | -9.436239259 |
| hsa04022 | cGMP-PKG signaling pathway | -9.75724 | 15.30069093 | -9.239350924 |
| hsa05143 | African trypanosomiasis | -9.66716 | 43.94719335 | -9.157467117 |
| hsa05216 | Thyroid cancer | -9.66716 | 43.94719335 | -9.157467117 |
| hsa04912 | GnRH signaling pathway | -9.56547 | 22.47990074 | -9.059813473 |
| hsa04664 | Fc epsilon RI signaling pathway | -9.24823 | 27.32850679 | -8.746581845 |
| hsa04140 | Autophagy - animal | -9.22919 | 16.47463175 | -8.731507193 |
| hsa04914 | Progesterone-mediated oocyte maturation | -9.20277 | 20.49638009 | -8.70901513 |
| hsa05211 | Renal cell carcinoma | -9.19627 | 26.93244147 | -8.706415414 |
| hsa04072 | Phospholipase D signaling pathway | -9.02291 | 15.6954262 | -8.536913012 |
| hsa04725 | Cholinergic synapse | -8.80396 | 18.50115725 | -8.321783913 |
| hsa04015 | Rap1 signaling pathway | -8.69992 | 12.16769231 | -8.221541364 |
| hsa04913 | Ovarian steroidogenesis | -8.63788 | 31.88325792 | -8.163263269 |
| hsa04611 | Platelet activation | -8.44522 | 16.85992556 | -7.974329463 |
| hsa05416 | Viral myocarditis | -8.12862 | 27.10076923 | -7.661421831 |
| hsa04910 | Insulin signaling pathway | -8.0634 | 15.26007861 | -7.599871873 |
| hsa04371 | Apelin signaling pathway | -8.00818 | 15.04050913 | -7.548280989 |
| hsa04928 | Parathyroid hormone synthesis, secretion and action | -7.6969 | 17.53149492 | -7.243675325 |
| hsa04920 | Adipocytokine signaling pathway | -7.69639 | 23.56588629 | -7.243675325 |
| hsa05332 | Graft-versus-host disease | -7.58211 | 33.18461538 | -7.132936715 |
| hsa04137 | Mitophagy - animal | -7.56578 | 22.58397436 | -7.120126573 |
| hsa04390 | Hippo signaling pathway | -7.54702 | 13.31611955 | -7.104850495 |
| hsa04940 | Type I diabetes mellitus | -7.51837 | 32.41288014 | -7.079664452 |
| hsa04930 | Type II diabetes mellitus | -7.33644 | 30.29899666 | -6.901169473 |
| hsa05014 | Amyotrophic lateral sclerosis | -7.19884 | 7.657988166 | -6.766978796 |
| hsa04211 | Longevity regulating pathway | -6.92209 | 18.2701815 | -6.493603788 |
| hsa04923 | Regulation of lipolysis in adipocytes | -6.81186 | 24.88846154 | -6.386724078 |
| hsa05012 | Parkinson disease | -6.60156 | 8.732793522 | -6.179759643 |
| hsa04261 | Adrenergic signaling in cardiomyocytes | -6.52417 | 12.38892308 | -6.105668972 |
| hsa04623 | Cytosolic DNA-sensing pathway | -6.50185 | 22.12307692 | -6.08662752 |
| hsa04929 | GnRH secretion | -6.46063 | 21.77740385 | -6.048656206 |
| hsa04810 | Regulation of actin cytoskeleton | -6.33083 | 9.5900494 | -5.922088953 |
| hsa04960 | Aldosterone-regulated sodium reabsorption | -6.27242 | 31.39085239 | -5.86688488 |
| hsa05204 | Chemical carcinogenesis - DNA adducts | -6.26444 | 20.1993311 | -5.862083651 |
| hsa04060 | Cytokine-cytokine receptor interaction | -6.19033 | 7.874315515 | -5.791137617 |
| hsa04670 | Leukocyte transendothelial migration | -6.18466 | 14.26356275 | -5.788594585 |
| hsa04310 | Wnt signaling pathway | -6.16914 | 11.12777522 | -5.776195531 |
| hsa04726 | Serotonergic synapse | -6.15893 | 14.13953177 | -5.769070824 |
| hsa04650 | Natural killer cell mediated cytotoxicity | -5.7776 | 12.41256606 | -5.39081212 |
| hsa04728 | Dopaminergic synapse | -5.75548 | 12.31853147 | -5.37174105 |
| hsa04613 | Neutrophil extracellular trap formation | -5.74761 | 9.780728745 | -5.366896834 |
| hsa04540 | Gap junction | -5.63823 | 15.83811189 | -5.260525947 |
| hsa04080 | Neuroactive ligand-receptor interaction | -5.3942 | 6.416914577 | -5.01947896 |
| hsa04061 | Viral protein interaction with cytokine and cytokine receptor | -5.31419 | 13.93753846 | -4.942426759 |
| hsa04934 | Cushing syndrome | -5.29213 | 10.49062035 | -4.923311661 |
| hsa05016 | Huntington disease | -5.12212 | 6.832126697 | -4.756233678 |
| hsa04152 | AMPK signaling pathway | -4.85834 | 11.61461538 | -4.495356291 |
| hsa04520 | Adherens junction | -4.84805 | 16.35861322 | -4.487950111 |
| hsa05330 | Allograft rejection | -4.66542 | 24.45182186 | -4.308187608 |
| hsa00980 | Metabolism of xenobiotics by cytochrome P450 | -4.6486 | 14.89053254 | -4.294215038 |
| hsa04114 | Oocyte meiosis | -4.64185 | 10.63934234 | -4.290299473 |
| hsa04610 | Complement and coagulation cascades | -4.46766 | 13.66425339 | -4.118919544 |
| hsa04550 | Signaling pathways regulating pluripotency of stem cells | -4.42744 | 9.746530393 | -4.081488652 |
| hsa05410 | Hypertrophic cardiomyopathy | -4.34807 | 12.90512821 | -4.004888811 |
| hsa04350 | TGF-beta signaling pathway | -4.25748 | 12.35597381 | -3.917061731 |
| hsa04672 | Intestinal immune network for IgA production | -4.22339 | 18.96263736 | -3.888430622 |
| hsa05030 | Cocaine addiction | -4.22339 | 18.96263736 | -3.888430622 |
| hsa04750 | Inflammatory mediator regulation of TRP channels | -4.17101 | 11.85164835 | -3.838755798 |
| hsa00330 | Arginine and proline metabolism | -4.15459 | 18.21900452 | -3.825030545 |
| hsa04916 | Melanogenesis | -4.10864 | 11.49961919 | -3.781754851 |
| hsa00480 | Glutathione metabolism | -3.93482 | 16.02015915 | -3.610590754 |
| hsa00140 | Steroid hormone biosynthesis | -3.84924 | 15.23228247 | -3.530276652 |
| hsa00590 | Arachidonic acid metabolism | -3.84924 | 15.23228247 | -3.530276652 |
| hsa04213 | Longevity regulating pathway - multiple species | -3.82172 | 14.9866005 | -3.505364054 |
| hsa05217 | Basal cell carcinoma | -3.79468 | 14.74871795 | -3.48091087 |
| hsa05031 | Amphetamine addiction | -3.64157 | 13.46622074 | -3.330376491 |
| hsa04270 | Vascular smooth muscle contraction | -3.53296 | 8.667623421 | -3.224337801 |
| hsa04723 | Retrograde endocannabinoid signaling | -3.33484 | 7.847713098 | -3.028763097 |
| hsa04970 | Salivary secretion | -3.16519 | 10.09966555 | -2.861639358 |
| hsa00380 | Tryptophan metabolism | -3.10153 | 16.59230769 | -2.80050007 |
| hsa04666 | Fc gamma R-mediated phagocytosis | -3.07894 | 9.579064235 | -2.780412048 |
| hsa04530 | Tight junction | -3.07415 | 6.872553482 | -2.778110354 |
| hsa04141 | Protein processing in endoplasmic reticulum | -3.05126 | 6.79217274 | -2.757695488 |
| hsa04640 | Hematopoietic cell lineage | -3.0458 | 9.385547786 | -2.754698834 |
| hsa04360 | Axon guidance | -2.93061 | 6.381656805 | -2.641954988 |
| hsa05034 | Alcoholism | -2.87851 | 6.21102427 | -2.592287802 |
| hsa04340 | Hedgehog signaling pathway | -2.73772 | 12.44423077 | -2.453920216 |
| hsa04730 | Long-term depression | -2.65167 | 11.61461538 | -2.370271407 |
| hsa04720 | Long-term potentiation | -2.51506 | 10.40114811 | -2.236058917 |
| hsa00982 | Drug metabolism - cytochrome P450 | -2.42668 | 9.678846154 | -2.150056295 |
| hsa03320 | PPAR signaling pathway | -2.37682 | 9.291692308 | -2.102557512 |
| hsa04971 | Gastric acid secretion | -2.36068 | 9.169433198 | -2.088773959 |
| hsa04612 | Antigen processing and presentation | -2.32909 | 8.934319527 | -2.059528562 |
| hsa00983 | Drug metabolism - other enzymes | -2.29838 | 8.710961538 | -2.031149111 |
| hsa04911 | Insulin secretion | -2.21111 | 8.103220036 | -1.946187778 |
| hsa05414 | Dilated cardiomyopathy | -2.07967 | 7.259134615 | -1.819347948 |
| hsa05150 | Staphylococcus aureus infection | -2.07967 | 7.259134615 | -1.819347948 |
| hsa04713 | Circadian entrainment | -2.06737 | 7.184298176 | -1.809331365 |
