# Supplementary material for: Exploring the mechanism of action of the combination of cinnamon and motherwort in the treatment of benign prostatic hyperplasia: A network pharmacology study
Source: Medicine (Baltimore). 2024 Apr 26;103(17):e37902. doi: 10.1097/MD.0000000000037902 (PMC11049697; doi:10.1097/MD.0000000000037902)
Supplement: Supplementary file 4 [file medi-103-e37902-s004.docx]

| BP |  |  |
| --- | --- | --- |
| pathway | enrichment | pvalue |
| response to hormone | 45.19542 | 1.57E+45 |
| cellular response to lipid | 40.14527 | 1.40E+40 |
| cellular response to organic cyclic compound | 38.61118 | 4.08E+38 |
| response to inorganic substance | 38.06816 | 1.17E+38 |
| cellular response to organonitrogen compound | 37.60801 | 4.06E+37 |
| cellular response to nitrogen compound | 37.23795 | 1.73E+37 |
| positive regulation of cell migration | 36.81669 | 6.56E+36 |
| positive regulation of cell motility | 36.09252 | 1.24E+36 |
| positive regulation of cellular component movement | 35.71358 | 5.17E+35 |
| positive regulation of locomotion | 35.65607 | 4.53E+35 |
| CC |  |  |
| pathway | enrichment | pvalue |
| membrane raft | 14.37082 | 2.35E+14 |
| membrane microdomain | 14.34777 | 2.23E+14 |
| transcription regulator complex | 10.2978 | 1.99E+10 |
| vesicle lumen | 9.787128 | 6.13E+09 |
| secretory granule lumen | 8.810158 | 6.46E+08 |
| cytoplasmic vesicle lumen | 8.761518 | 5.77E+08 |
| caveola | 8.586894 | 3.86E+08 |
| RNA polymerase II transcription regulator complex | 8.057186 | 1.14E+08 |
| plasma membrane raft | 7.478194 | 3.01E+07 |
| protein kinase complex | 7.217688 | 1.65E+07 |
| MF |  |  |
| pathway | enrichment | pvalue |
| kinase binding | 21.85724 | 7.20E+21 |
| protein kinase binding | 19.91274 | 8.18E+19 |
| DNA-binding transcription factor binding | 19.54042 | 3.47E+19 |
| transcription factor binding | 19.34437 | 2.21E+19 |
| protein homodimerization activity | 17.77316 | 5.93E+17 |
| RNA polymerase II-specific DNA-binding transcription factor binding | 17.52035 | 3.31E+17 |
| protein domain specific binding | 13.62805 | 4.25E+13 |
| protease binding | 13.53479 | 3.43E+13 |
| ubiquitin-like protein ligase binding | 13.38113 | 2.41E+13 |
| protein kinase activity | 13.32545 | 2.12E+13 |
| KEGG |  |  |
| pathway | enrichment | pvalue |
| Pathways in cancer | 38.59525 | 2.70E+69 |
| AGE-RAGE signaling pathway in diabetic complications | 45.23883 | 1.52E+47 |
| Fluid shear stress and atherosclerosis | 39.47645 | 1.72E+44 |
| Lipid and atherosclerosis | 34.57577 | 1.27E+43 |
| Prostate cancer | 39.73759 | 2.15E+39 |
| PI3K-Akt signaling pathway | 28.15293 | 3.34E+38 |
| MAPK signaling pathway | 26.61683 | 3.20E+33 |
| Proteoglycans in cancer | 27.95637 | 6.20E+31 |
| IL-17 signaling pathway | 34.07376 | 5.97E+31 |
| HIF-1 signaling pathway | 30.09013 | 2.37E+28 |
| Cellular senescence | 27.37585 | 1.67E+28 |
| TNF signaling pathway | 29.66721 | 1.28E+28 |
| Apoptosis | 28.11035 | 8.52E+27 |
| Endocrine resistance | 30.25595 | 3.72E+27 |
| Relaxin signaling pathway | 27.55255 | 5.26E+26 |
| FoxO signaling pathway | 23.31866 | 3.33E+21 |
| EGFR tyrosine kinase inhibitor resistance | 25.22514 | 1.66E+20 |
| Estrogen signaling pathway | 20.07658 | 8.65E+17 |
| VEGF signaling pathway | 19.3987 | 1.24E+13 |
| Regulation of actin cytoskeleton | 8.369691 | 2142058 |
